# Understanding the role of immune-mediated inflammatory disease related cytokines interleukin 17 and 23 in pregnancy: A systematic review

**DOI:** 10.1016/j.jtauto.2025.100279

**Published:** 2025-02-07

**Authors:** Aniek Plug, Liana Barenbrug, Bart G.J. Moerings, Elke M.G. de Jong, Renate G. van der Molen

**Affiliations:** a Department of Laboratory Medicine, Laboratory of Medical Immunology, Radboud University Medical Center (Radboudumc), Nijmegen, the Netherlands; b Department of Dermatology, Radboud University Medical Center (Radboudumc), Nijmegen, the Netherlands

**Keywords:** Immune-mediated inflammatory disease, Psoriasis, Interleukin 17, Interleukin 23, Pregnancy, Trophoblast

## Abstract

**Background:**

Pregnancy requires a careful immune balance between tolerance for the semi-allogenic fetus and protection against pathogens. Women with immune-mediated inflammatory diseases (IMIDs), where the interleukin (IL)-23/IL-17 axis plays an important role, often experience changes in disease severity during pregnancy. These changes and the association between disease flares and pregnancy complications, suggests a role for IL-17 and IL-23 in pregnancy.

**Methods:**

We systemically searched PubMed, EMBASE, and Web of Science (March 2024), to assess the role of IL-17 and IL-23 in pregnancy-related *in vitro* assays, animal or human studies.

**Results:**

Eighty articles (8 *in vitro*, 11 animal and 61 human studies) were included. Seventy-one studies reported on IL-17 and 16 studies on IL-23. *In vitro* trophoblast proliferation, migration and invasion was increased in the presence of IL-17, but impaired with IL-23. IL-17 levels were increased in animal models for pregnancy complications. In humans, IL-17 levels seemed to be increased in pregnant women versus non-pregnant women. Additionally, elevated IL-17 levels were associated with pregnancy complications. Although similar trends were found for IL-23, data were limited.

**Conclusions:**

We identified a large, but heterogenic, body of evidence for a significant role of IL-17 in all stages of pregnancy: while an excessive increase seemed to be associated with complications. The limited number of studies prevents firm conclusions on the role of IL-23. Future research is needed to find biomarkers for patients with IMIDs to predict the effect of possible disease flares on pregnancy, and the effect of therapeutic inhibition of IL-17 or IL-23.

## Highlights

• An increase in IL-17 seems to be required to establish and maintain pregnancy.
• Excessive IL-17 levels were associated with pregnancy complications.
• A well-controlled balance of IL-17 seems to be required for a successful pregnancy.
• The limited number of studies prevents firm conclusions on the role of IL-23 in pregnancy.

## Graphical abstract

**Figure:**
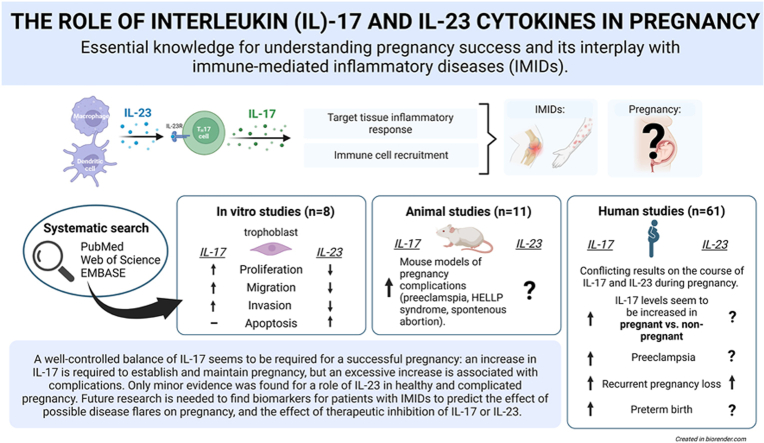

## 1 Introduction

Pregnancy encompasses a unique but precise immunological balance: a pro-inflammatory environment is essential for proper implantation and parturition, whereas a tolerogenic environment for the semi-allogenic fetus is needed throughout pregnancy to enable normal placentation and fetal development [1]. Thus, pre-existing disturbances of the immune system due to immune-mediated inflammatory disorders (IMIDs) may have a significant impact on pregnancy outcomes, while in turn, the immunological alterations of pregnancy may affect maternal IMIDs disease status. Unpredictable fluctuations of severity of IMIDs in pregnancy with both disease amelioration as well as deterioration, depending on disease type and patient-specific underlying mechanisms, are reported [2].

Cytokines are important immunomodulators attributing to the systemic nature of all IMIDs, maintaining a constant state of inflammation through positive-feedback loops. One such IMID is psoriasis, a chronic disease characterized by aberrant proliferation and differentiation of epidermal keratinocytes, resulting in the formation of erythematous, scaly plaques on the skin [3]. Psoriasis affects approximately 2–3% of the global population, with variable clinical manifestations and disease severity, and, most importantly in the current context, peak onset during reproductive age [4]. The pivotal role for the interleukin-23 (IL-23) and interleukin-17 (IL-17) cytokine axis in the pathogenesis of psoriasis has been extensively studied in recent years [5]. The IL-23/IL-17 axis is a critical signaling pathway in the immune system, driving inflammatory responses and chronic inflammation: IL-23 stimulates the differentiation and activation of Th17 cells, which produce IL-17 and other pro-inflammatory cytokines, while IL-17 orchestrates the recruitment of immune cells and amplifies the inflammatory cascade within target tissues [6]. The IL-17 family of pro-inflammatory cytokines includes six members: IL-17A to IL-17F, with IL-17A and IL-17F being produced by Th17 cells that play a role in the IL-23/IL-17 axis [7]. IL-23, a cytokine of the IL-12 family, is composed of two subunits: IL-12B (IL-12p40) and IL-23A (IL-23p19) [8]. Dysregulation of the IL-23/IL-17 axis in psoriasis drives the chronic inflammation underlying the disorder [4,5]. Conversely, the role of this axis in pregnancy remains poorly understood. Approximately 55 % of psoriasis patients experience (partial) disease remission during pregnancy and 10–20% experience exacerbations, while disease status returns to pre-pregnancy severity after delivery in 40–90% of women [2]. Additionally, disease exacerbations might negatively affect pregnancy outcomes [9]. These observations suggest that pregnancy-related immune alterations can somehow interfere with the immunological pathway of psoriasis, possibly via the IL-23/IL-17 axis.

Biologics specifically targeting and inhibiting the IL-23/IL-17 axis, such as secukinumab (IL-17A inhibitor) and ustekinumab (IL-12p40 inhibitor) have recently emerged as a new therapeutic strategy for psoriasis and other IL-17/IL-23 driven IMIDs, showing substantial clinical efficacy and safety profiles [10]. However, the effects of this cytokine interference on the immunological balance during pregnancy and thus the female reproductive system and pregnancy outcome, as well as maternal disease status during pregnancy, are largely uninvestigated. Safety data on the use of these biologics during pregnancy is limited to case reports or first trimester exposure only [[11], [12], [13], [14], [15]]. A recent meta-analysis reported no correlation between biologic use and miscarriage or congenital abnormalities in psoriatic women, but significant limitations hamper the ability to draw firm conclusions; included studies were often confounded by tobacco use and comorbidities, amongst others [16]. Additionally, there was a lack of studies on the more novel IL-17 and IL-23 inhibitors brodalumab, risankizumab or bimekizumab, and the authors raise the possibility of publication and reporting bias [16].

Understanding the intricate interplay of the IL-17 and IL-23 cytokines in the context of pregnancy is essential for unraveling the underlying immunological mechanisms that govern pregnancy success. In addition, this will help optimize the management of psoriasis and other inflammatory diseases in pregnant women, ensuring both maternal well-being and fetal health. For this objective, we performed a systematic review on the role of IL-17 and IL-23 in pregnancy and pregnancy-related tissues.

## 2 Methods

### 2.1 Search strategy

A systematic literature search of PubMed, EMBASE and Web of Sciences was conducted on March 14, 2024 to identify eligible peer-reviewed research articles (Appendix A). The search was performed using the following keywords: decidua, placenta, preconception, pregnancy, prenatal, trophoblasts, uterus, interleukin-17 and interleukin23 (including interleukin-25 (i.e., IL-17E) and CTLA-8 (i.e., IL-17A)). This study was registered in PROSPERO (CRD42023487972) and reported following the PRISMA guidelines [17]. Of note: although only IL-17A and IL-17F are primarily known to be involved in the IL-23/IL-17 axis, other members of the IL-17 family of cytokines have been found to contribute to the inflammation in IMIDs as well [18]. Additionally, the IL-17 inhibitor brodalumab blocks the IL-17 receptor and therefore all members of the IL-17 family [18]. For this reason, we did not exclude IL-17B, C, D and E from the search strategy.

### 2.2 Eligibility criteria

Studies were included if they reported on the effect of IL-17 and/or IL-23 (including administration or inhibition) in pregnancy related tissues or cells, or animal or human pregnancy.

For *in vitro* studies, studies were included when reporting the effects of IL-17 or IL-23 treatment (plus possible inhibition) on reproductive tissue cell functions relating directly to successful pregnancy, i.e., trophoblast proliferation, invasion, migration and apoptosis, and stromal decidualization. Progesterone secretion was considered a too indirect outcome related to pregnancy: studies solely reporting trophoblast progesterone secretion were therefore excluded. Studies measuring *in vitro* secretion of IL-17 or IL-23 in pregnancy related tissues or cells were included, but studies measuring *in vitro* IL-17 or IL-23 secretion/expression of PBMCs isolated *in vivo* from pregnant women were excluded due to the limitations of comparing highly variable isolation and culture protocols.

Animal studies were included when performed in laboratory animals and aimed to understand human pregnancy and human pregnancy complications, while veterinary studies in strictly agricultural settings in e.g., cattle were excluded. Anti-IL-17 or anti-IL-23 biologics toxicology studies in animals were excluded. For animal and human studies, studies concerning any maternal disease were excluded, with exception of direct pregnancy related complications. For animal studies, included complications were pregnancy loss, maternal immune activation (MIA), preeclampsia (PE) (by means of the reduced uterine perfusion pressure (RUPP) model), and hemolysis, elevated liver enzymes and low platelets (HELLP) syndrome. For human studies, included complications were gestational hypertension (GH), placental or cervical insufficiency, PE, HELPP syndrome, preterm birth or labor (PTB or PTL), and (unexplained) recurrent pregnancy loss (RPL) (including its synonyms spontaneous abortion or miscarriage). Studies measuring IL-17 or IL-23 only in women with previous pregnancy complications but who were not pregnant at the time of measurement were excluded. Only soluble IL-17 and IL-23 measurements were considered relevant outcomes for this review, i.e., studies reporting the number of IL-17- or IL-23-expressing cells or intracellular concentrations as measured by flow cytometry or similar methods, were excluded. However, studies assessing IL-17 or IL-23 expression in pregnancy related tissue (i.e., placenta or decidua) were included. To minimize confounding of immunological fluctuations associated with birth, studies measuring IL-17 or IL-23 during or post-partum were excluded. Studies focused on fertility in non-pregnant subjects were excluded and relating to this, studies reporting on assisted reproductive technologies (ART) were only included when data on final pregnancy outcomes were available. ART studies reporting solely on ART success-rate were thus excluded.

Studies from the same authors were carefully examined for overlap in study cohort. All included studies were assessed by two reviewers (AP, LB) with one reviewer available for second opinions (RM). Discrepancies were solved through consensus. Studies in languages other than English, *in silico* studies, reviews, letters or case reports were excluded. No restrictions by date of publication were applied.

### 2.3 Data extraction and quality assessment

Separate data extraction tables were constructed for *in vitro*, animal, and human studies. For *in vitro* studies, the following data were extracted: author, year of publication, cell type, intervention (treatment, dosage, and timing), outcome measures and techniques, and their results with p-values. For animal studies, the study characteristics (author, year of publication, size experimental group and size control group), animal characteristics (species, model, gestational age (GA) at intervention and at measurement), intervention and dosage, relevant outcome measures and techniques, and significant results were extracted. For human studies, the following data were extracted: study characteristics (author, year of publication, definition and size case group, definition and size control group, before or after therapy), outcome measures (biofluid/tissue source, measurement technique, GA at time of measurement), and their results with p-values. IL-17 or IL-23 levels that were only reported in graphs but not in-text were manually extracted using the free online WebPlotDigitizer tool. Here, the middle of the horizontal lines of the ticks of the y-axis and the (error) bars were used for axis calibration and value extraction, respectively. All values were extracted from the graphs with three significant digits.

Risk of bias and quality of evidences of studies including human subjects were assessed with respectively ROBINS-I tool and Quality Rating Scheme for studies and Other evidence, a modification from the Oxford Centre for Evidence-based Medicine [19].

### 2.4 Data visualization

To visualize the current knowledge of IL-17 and IL-23 levels throughout healthy pregnancy and in pregnancy complications, we constructed line graphs. For comparability reasons, only studies that reported mean serum levels measured by ELISA were included and graphs were only constructed when >3 studies could be included. Standard deviations/errors were omitted from the final graphs to ensure readability. The graphs were constructed in GraphPad (Version 10.1.2.).

## 3 Results

### 3.1 Study characteristics

**Fig. 1:**
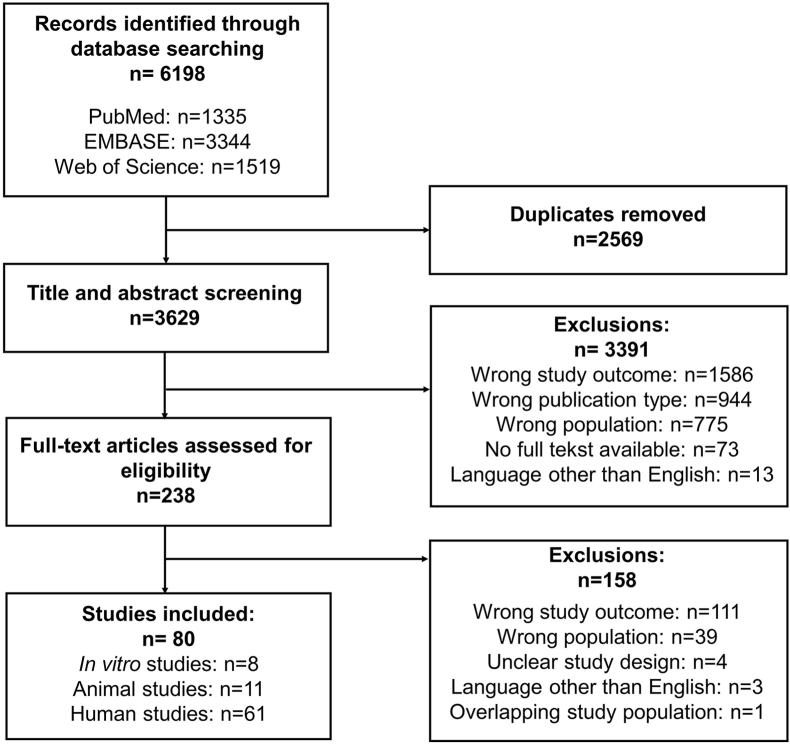
PRISMA flow diagram of assessment of identified studies.

We identified 3629 articles after exclusion of duplicates, which were screened for title and abstract. We assessed 238 full-text articles for eligibility, resulting in 80 final articles of which 8 were *in vitro* studies, 11 were studies in animals, and 61 were studies in humans (Fig. 1). The following sections describe general study characteristics and outcomes of the included articles.

### 3.2 Quality assessment

We assessed all studies involving human subjects for risk of bias using the ROBINS-I tool (Appendix B). Twenty-eight studies were considered to have low risk of bias, 30 to have moderate risk of bias, and 6 to have serious risk of bias. The risk of bias was increased when studies did not take into account confounding factors (i.e., GA), the study population was not clearly described, or results were only showed for subgroups. Using the Quality Rating Scheme for Studies and Other Evidence, 45 studies were rated with a “4 - case series or cross-sectional study”, 9 with “3 - case control or retrospective study”, and 8 with “2 - well-designed controlled trial without randomization or prospective comparative cohort trial” (Appendix C).

### 3.3 In vitro studies

#### 3.3.1 In vitro study characteristics

Seven out of the 8 included *in vitro* studies focused on IL-17 [[20], [21], [22], [23], [24], [25], [26]] while only one study reported on culture with IL-23 [27]. Details of all *in vitro* studies can be found in Appendix D. One study used primary first trimester trophoblasts [20], and five studies used commercially available trophoblast cell lines: HTR/SVneo [23,24], JEG-3 [21] or Swan71 [22] cells (the latter forming blastocyst-like spheroids). One study used placental explants from humans [25] and one study from mice [26]. Four studies used recombinant IL-17 [21,22], IL-25 (IL-17E) [23] or IL-17A [20] to treat the cells, while one study used IL-17 overexpressing plasmid transfection [24]. The IL-25 study included an anti-IL-25 neutralizing antibody condition (with or without concomitant IL-25 treatment), while the plasmid study inhibited IL-17 by small-interfering RNA (si-RNA).

#### 3.3.2 The role of IL-17 in the functioning of reproductive tissue derived cells

A dose-dependent increase in invasion and proliferation, but no effect on apoptosis was observed following recombinant IL-17A treatment of healthy first trimester trophoblasts [20]. IL-17 was found to increase invasion, attachment and migration of JEG-3 [21], Swan71 [22] and HTR-8/SVneo cells [23,24]. In the presence of IL-17 an increased proliferation was found in HTR-8/SVneo cells [23,24], but not in JEG-3 cells [21]. In HTR-8/SVneo cells, IL-17 inhibition by culture with IL-25 neutralizing antibody neutralized the observed effects [23] and IL-17 inhibition by transfection of si-IL-17 showed opposing effects to IL-17 transfection for all outcomes [24].

In human placental explants higher IL-17 levels were found in the supernatant of early placental explants than term placental explants [25]. In mouse placental explants of abortion prone mice IL-17 were not statistically significant different compared to non-abortion prone mice [26].

#### 3.3.3 The role of IL-23 in the functioning of reproductive tissue derived cells

In a study using HTR-8/SVneo trophoblasts, treatment of the cells with recombinant IL-23 significantly inhibited cell proliferation, migration and invasion, while apoptosis was increased [27].

### 3.4 Animal studies

#### 3.4.1 Animal study characteristics

Three of the 11 identified animal studies used a rat model [[28], [29], [30]], while the other 8 studies used a mouse model [26,[31], [32], [33], [34], [35], [36], [37]]. Details of all animal studies can be found in Appendix E. IL-17 was assessed by 9 studies [26,[28], [29], [30],[32], [33], [34], [35], [36]], IL-23 by one study [37], and one study assessed both IL-17 and IL-23 [31]. Five studies used an uncomplicated pregnancy model, and the six other studies included pregnancy complications (MIA [35,36], abortion [[33], [34]], RUPP [29], HELPP [30]).

#### 3.4.2 The role of IL-17 in murine and rat pregnancy

One study found no IL-17 expression in mid-pregnancy placenta of normal pregnant mice [26]. IL-17 levels in the placenta were reported to remain constant from mid to late gestation [31], while another study reported a statistically significant increase of serum IL-17 levels from mid to late gestation [32].

IL-17 seems to have an effect on pregnancy complications. One study found significantly increased maternal mean arterial pressure (MAP), increased total and cytolytic natural killer (NK) cells in circulation and placenta, increased placental inflammatory cytokines and increased placental reactive oxygen species (ROS), while vascular function in uterine arteries and fetal and placental weight were decreased upon chronic recombinant IL-17A/F infusion during gestational days (GD) 12–19 in normal pregnant rats [28]. Conversely, IL-17 inhibition by chronic IL-17 receptor C infusion from GD14-19 in pregnant RUPP rats significantly prevented placental ischemia-induced increases in MAP, total and cytolytic placental NK cells, levels of various cytokines and placental ROS induction while vascular function and fetal and placental weight were rescued compared to control non-treated RUPP rats [29]. Also, plasma IL-17 levels were statistically significant increased in HELLP rats compared to healthy pregnant rats [30]. A single recombinant IL-17 injection at embryonic day 4 induced abortion in normal pregnancy, while anti-IL-17 antibody injection prevented fetal loss in the abortion prone model [33]. Moreover, abortion prone mice had higher IL-17A levels in serum and decidua compared to non-abortion prone mice [34]. The two studies with a MIA mouse model demonstrated increased IL-17 levels [35,36], although one study did not found a statistically significant different [36].

#### 3.4.3 Role of IL-23 in murine pregnancy

No expression of IL-23 was found in murine placenta [37]. IL-23 serum levels remained constant from mid to late gestation in mice [31].

### 3.5 Human studies

#### 3.5.1 Characteristics of human studies

A total of 61 studies were identified reporting on IL-17 (n = 58) and/or IL-23 (n = 13) in healthy and complicated human pregnancy. Details of all human studies on IL-17 and IL-23 studies can be found in Appendix F and G respectively.

For IL-17, nine studies compared single serum or plasma levels of IL-17 in healthy pregnant women with those in non-pregnant women [[38], [39], [40], [41], [42], [43], [44], [45], [46]], while four studies measured serum IL-17 levels longitudinally during healthy pregnancy [[47], [48], [49], [50]]. In addition, two studies assessed IL-17 levels in amniotic fluid [51,52], and one study determined IL-17 levels in the placenta [53]. Regarding pregnancy complications, 33 studies reported on PE [41,42,[44], [45], [46],^48^,[54], [55], [56], [57], [58], [59], [60], [61], [62], [63], [64], [65], [66], [67], [68], [69], [70], [71], [72], [73], [74], [75], [76], [77], [78], [79], [80]], 13 on RPL [39,53,[81], [82], [83], [84], [85], [86], [87], [88], [89], [90], [91]], and 4 on PTB [[92], [93], [94], [95]].

For IL-23, three studies compared single serum or plasma levels in healthy pregnant women with levels in non-pregnant women [[43], [44], [45]] and one study determined IL-23 levels in the placenta [96]. Regarding pregnancy complications, 7 studies reported on PE [44,45,65,66,80,97,98] and 5 on RPL [81,[84], [85], [86], [87]].

#### 3.5.2 IL-17 in healthy human pregnancy

IL-17 is weakly to moderately expressed in cytotrophoblasts and moderately to strongly expressed in syncytiotrophoblasts, in healthy term placentas [53].

The results of studies comparing plasma or serum IL-17 levels during the first trimester to levels in non-pregnant women, were conflicting; one study reported higher plasma IL-17 levels in non-pregnant women [38], while another study reported no difference in serum IL-17 levels between non-pregnant women compared to first trimester pregnancy [39]. IL-17 serum and plasma levels were generally found to be elevated in the third trimester of pregnancy [[40], [41], [42], [43], [44], [45]], except for one study [46], compared to levels in non-pregnant women.

**Fig. 2:**
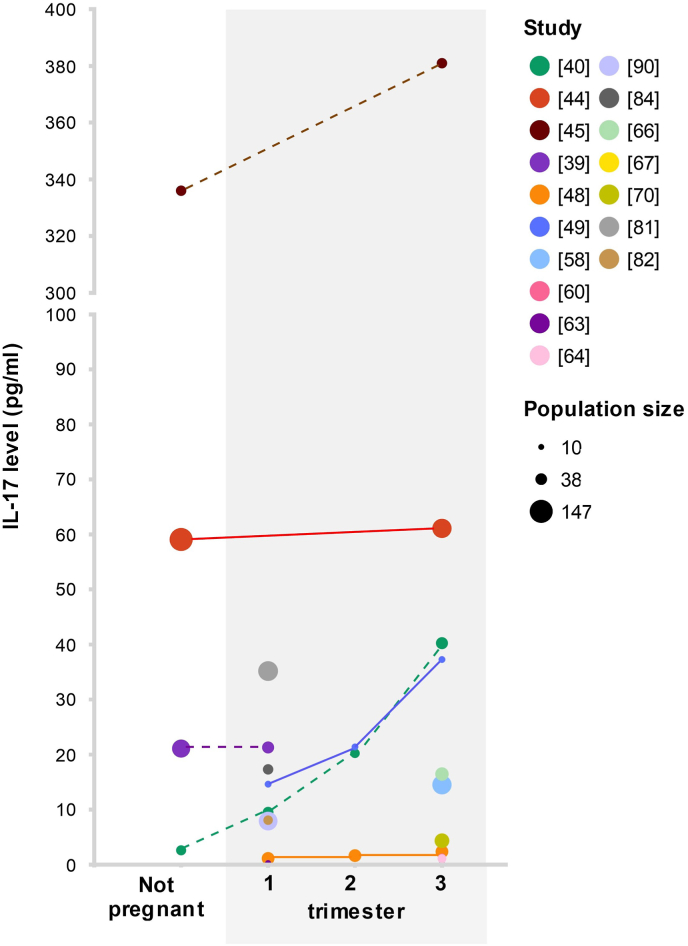
**Visualization of mean interleukin-17 (IL-17) levels reported by the studies included in this review, as measured by ELISA in serum of healthy pregnant women compared to non-pregnant controls**. Dotted lines indicate samples from the different treatment groups were taken from different women, while solid lines represent longitudinal measurements within the same women over time.

There were conflicting results on the course of serum IL-17(A) when measured longitudinally in pregnancy (Fig. 2): with studies describing a decrease [47], a significant increase [48,49], fluctuations in levels [50], or no statistically significant changes over time [38] throughout the three trimesters.

When measured in amniotic fluid, IL-17 was found to be present in statistically significantly lower levels than in serum [51]. No significant difference was found in amniotic fluid IL-17 levels at mid-trimester compared to term [52].

#### 3.5.3 IL-17 levels in human pregnancy complications

##### 3.5.3.1 IL-17 in preeclampsia (PE)

In the first trimester no differences in serum IL-17 levels were found in women with PE compared to healthy pregnant women [54,55].

In the second trimester an increased plasma IL-17 levels in women with PE compared to healthy pregnant women were found in two studies [56,57], while one study reported no statistically significant differences [59].

**Fig. 3:**
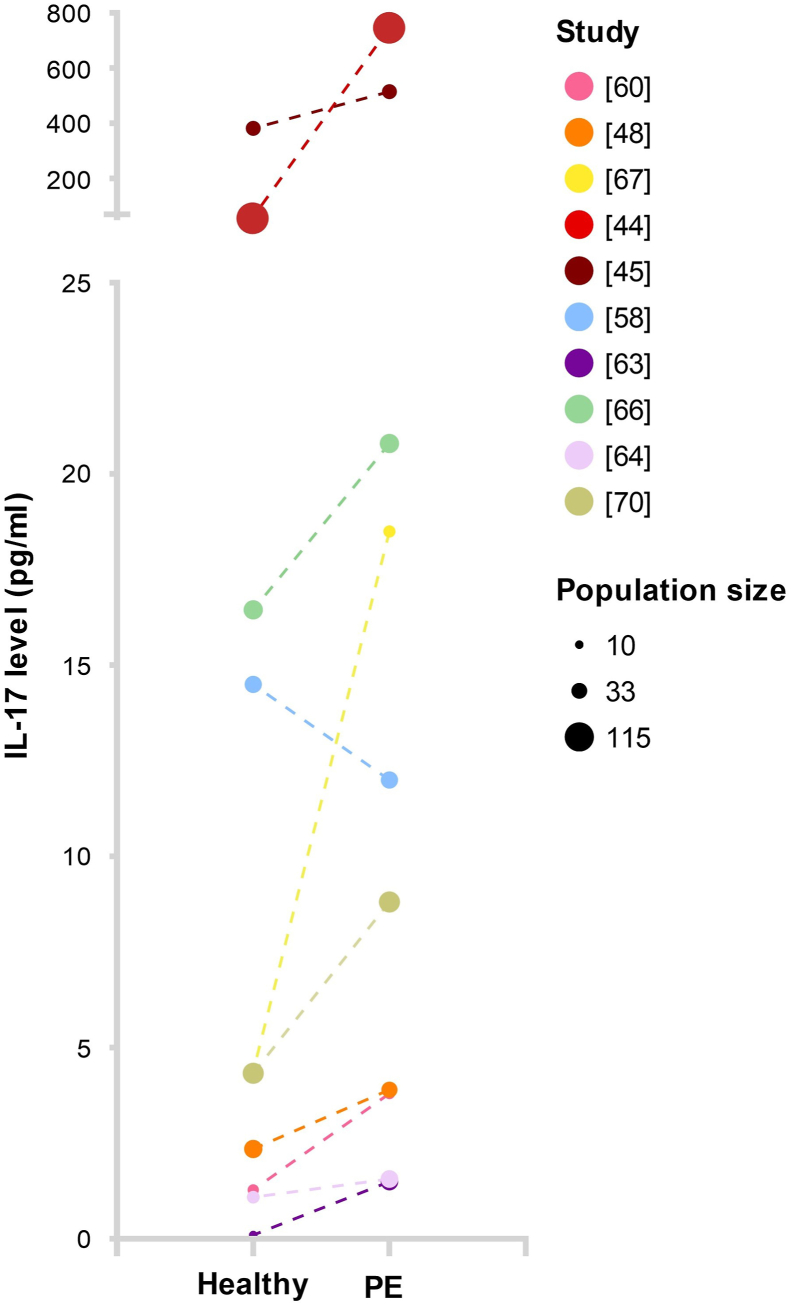
**Visualization of mean interleukin-17 (IL-17) levels reported by the studies included in this review, as measured by ELISA in serum of women with preeclampsia (PE) compared to healthy pregnant controls.** Dotted lines indicate samples from the different groups were taken from different women.

In the third trimester, most studies found statistically significant increased serum or plasma IL-17 levels in women with PE compared to healthy pregnant women [[44], [45], [46],^48^,^60^,^64^,^65^,^67^,^69^,[70], [71],^74^,^75^,^78^,^79^] (Fig. 3). However, some studies found no statistically significant differences in serum and plasma IL-17 levels between women with PE and healthy women [42,58,59,[61], [62], [63],^66^,^68^,^72^,^73^,^76^]. Two studies reported statistically significant decreased plasma IL-17 levels in women with PE [41,77].

In placental blood IL-17 levels and expression of IL-17 in the placenta were elevated in women with PE [58]. The expression of IL-17 in the decidua was not different between women with PE compared to healthy pregnant women [80].

##### 3.5.3.2 IL-17 in recurrent pregnancy loss (RPL)

Most studies reported on unexplained RPL (uRPL). Serum IL-17 levels were increased in uRPL patients compared to serum levels in first trimester healthy pregnant women [81,82,84], although not all studies were statistically significant [39]. One study studied the differences between pregnant women with a history of uRPL and healthy pregnant women both resulting in live births, but still found increased levels of IL-17 during the first trimester in women with a history of uRPL compared to healthy pregnant women [83]. Also, in decidual tissue of patients with uRPL IL-17 levels were increased compared to healthy pregnant women [[84], [85], [86], [87]]. Finally, a significant downregulation of IL-25 (i.e., IL-17E) gene expression was observed in trophoblastic tissue obtained from uRPL patients compared to elective abortion controls [88]. Two studies did not indicate if the RPL was unexplained. One of these studies showed a decrease in serum IL-17 levels in patients with RPL [90]. Another study showed decidual IL-17 expression in RPL patients, but no IL-17 expression in decidua of healthy women [91]. IL-17 was expressed in cyto-, syncytio-, and extravillous trophoblasts of pregnancy loss placentas [53]. However, this study did not report the number of previous pregnancy losses. One study assessed patients undergoing ART with a pregnancy resulting in pregnancy loss to pregnancies resulting in live birth; serum lL-17 levels were higher in cases of pregnancy loss compared to live birth [89].

##### 3.5.3.3 IL-17 in preterm birth (PTB)

Serum IL-17 and placental IL-17 in the third trimester of pregnancy was found to be significantly increased in women who eventually delivered preterm compared to those who delivered at term [[92], [93]]. Similarly, significantly increased IL-17 levels were found in second trimester amniotic fluid of women that ended up having a PTB compared to women that went on to have a term delivery [94]. When measured longitudinally in cervico-vaginal fluid, this effect was further observed as a non-significant positive association of IL-17 protein and PTB [95].

#### 3.5.4 IL-23 levels in healthy human pregnancy

IL-23 is expressed in decidual cells, glandular epithelium, cyto- syncytio- and extravillous trophoblasts [96]. The studies comparing serum or plasma IL-23 levels in pregnant women compared to non-pregnant women reported conflicting results: one study reported a significant decrease [45], one an increase [43] and one similar levels between pregnancy and controls [44]. Though, the latter two studies did not provide a significance level.

#### 3.5.5 IL-23 levels in human pregnancy complications

##### 3.5.5.1 IL-23 in preeclampsia (PE)

**Fig. 4:**
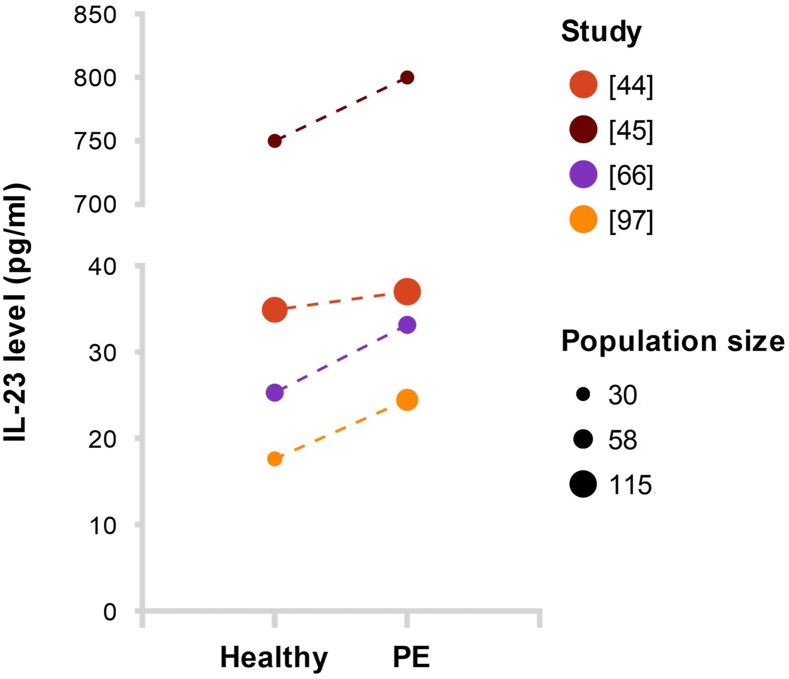
**Visualization of mean interleukin-23 (IL-23) levels reported by the studies included in this review, as measured by ELISA in serum of women with preeclampsia (PE) compared to healthy pregnant controls**. Dotted lines indicate samples from the different groups were taken from different women.

Most studies reported no significant differences between PE and normotensive controls [44,45,65,66] and one reported elevated levels in PE compared to healthy pregnant controls [97] (Fig. 4). Placental IL-23 mRNA expression was significantly increased in the chorionic layer, but not the decidua of PE placentas compared to healthy placentas [80], and a significantly higher expression was found in the decidua basalis, but not parietalis of both early- and late-onset placentas versus controls [98].

##### 3.5.5.2 IL-23 in recurrent pregnancy loss (RPL)

All studies reported on uRPL. Consistent with the findings on IL-17 levels in uRPL, IL-23 protein levels in serum and decidua were significantly higher in pregnant women with uRPL compared to healthy pregnant women [81,[84], [85], [86]]. Decidual mRNA levels were comparable between uRPL patients and healthy controls [86,87].

## 4 Discussion

We performed a systematic literature review to investigate what is known about the role of IL-17 and IL-23 in pregnancy and identified 80 studies divided over *in vitro* studies, animal studies and human studies. We were able to systematically and uniformly report all available data for optimal comparability and stacking of evidence.

*In vitro* studies on IL-17 provide strong evidence for a beneficial effect of IL-17 on trophoblast proliferation and invasion with some evidence for increased migration and decidualization, and decreased apoptosis. This is reflected by elevated IL-17 serum levels in human uncomplicated pregnancies compared to non-pregnant controls. On the other hand, IL-17 infusion/injection in pregnant animals resulted in negative effects that were rescued by IL-17 inhibition, several pregnancy complications (PE, HELLP syndrome, spontaneous abortion in mice and PTB, PE, uRPL in humans) were associated with elevated IL-17. Although a certain increase in IL-17 seems to be required for successful implantation and healthy pregnancy maintenance, these data suggest that an excessive increase might be detrimental. Importantly, this increase of IL-17 in healthy pregnancy compared to non-pregnancy is not self-evident: absolute levels and effect size differ highly between studies, and non-significant and opposite trends have also been reported.

In pregnancy complications, the question remains whether this excessive increase in IL-17 a cause or a consequence of the complication is. Most PTB studies were performed prospectively, providing odds ratios that ranged from 1.30 to 2.62 and clear evidence that elevated IL-17 levels during pregnancy are associated with and might be causal for PTB [[92], [93], [94], [95]]. However, studies on uRPL were never conducted prospectively and none of the identified studies reported on pre-pregnancy IL-17 levels in relation to pregnancy complications. Although we have identified prospective studies that showed increased IL-17 already in the second trimester of pregnancies that turn out to be PE pregnancies, placentation already starts at day 10 after conception and improper placental development thus may already occur in early trimesters. First or second trimester prospective studies do not hold enough power to confirm or reject increased IL-17 levels as a causal factor for PE. IL-17A is a known mediator of general hypertension with some early signs pointing towards beneficial effects of biologics in reducing hypertension [99]. This suggests that the use of the anti-IL-17 biologic could normalize excessive IL-17 levels and hereby potentially be protective against PE, but research is required to test this hypothesis.

The number of studies on IL-23 in pregnancy was much lower than on IL-17, possibly because IL-23 is more upstream, while IL-17 is a downstream effector cytokine in the IL-17/IL-23 axis, making the latter more relevant in examining direct physiological effects. *In vitro*, IL-23 inhibited trophoblast functioning and promoted apoptosis, suggesting a need for decreased IL-23 in successful pregnancy and thus beneficial effects of IL-23 inhibition. Human studies assessing IL-23 levels in pregnancy compared to non-pregnancy found conflicting results. Regarding pregnancy complications, IL-23 was associated with uRPL but less with PE. However, the small number of articles limit the ability to make strong conclusions on the role of IL-23 in pregnancy and pregnancy complications.

It is important to note that studies measuring IL-17 or IL-23 during or after third trimester vaginal delivery were excluded from this review to minimize confounding effects of immunological fluctuations occurring during the delivery. Two of the included studies obtained plasma/serum levels shortly prior to natural delivery, but their reported values are not markedly different from samples obtained earlier in the third trimester [42,77].

The IL-17 family comprises six distinct subtypes (A,B,C,D,E,F) [100,101]. The majority of the studies included in this review, particularly those involving human subjects, reported the use of IL-17A. Consequently our findings are primarily based on the observed effects of this specific subtype. However, numerous studies did not specify the subtype of IL-17 used. Especially subtypes A and F are associated with IMIDs like psoriasis [100,101]. Although A and F share overlapping functions, they also exhibit distinct functional differences [100,101]. Furthermore, their formation as either homodimer (AA, FF) or heterodimer (A/F), could potentially have differential effects [102]. Due to the lack of detailed reporting on IL-17 subtypes we were unable to assess potential differences in the effects of various IL-17 subtypes on pregnancy.

The heterogenous nature of underlying immune disturbances including fluctuations in cytokines such as IL-17 and IL-23 among IMID patients not only requires personalized therapeutic management [103], but also complicates prediction of reproductive outcomes and maternal disease fluctuations with or without biologic use during pregnancy, highlighting the need for predictive biomarkers for these patients. Within the framework of this review, it is important to answer the following questions: What are the levels of IL-17 and IL-23 in healthy individuals versus individuals with IMIDs, both outside pregnancy and during pregnancy, and how do these levels change with the use of biologic? As IL-17 and IL-23 are merely inhibited upon biologic use, protein and mRNA levels itself are not expected to change and therefore assays that specifically measure biologically active IL-17 or IL-23 could be a valuable measurement tool. Advanced *in vitro* models such as 3D organoids or co-cultures may assist in obtaining more detailed (molecular) mechanistic insights of the effects of IL-17 and IL-23 in pregnancy, building on the solely functional trophoblast assays that have been the focus of *in vitro* research thus far. *In vivo,* a novel tool in reproductive immunology is the use of menstrual blood [104]. Immune profiling of menstrual blood reveals the pre-pregnancy uterine immune environment that could provide insights into local optimal IL-17 and IL-23 levels for successful pregnancy, as well as more relevant reproductive effects of biologic-mediated inhibition as opposed to peripheral blood [105]. While this review focused on maternal pregnancy complications, information on transfer of the cytokines (and their inhibiting biologics) between mother and fetus by, for example, *ex vivo* placental perfusion studies would be needed to answer the question to what extent the maternal systemic inflammation translates to and affects the developing fetus.

Limitations of several studies in this review include lack of information on specific gestational age at measurement, medication use, IL-17 subtype, and number of samples below the detection limit. This may have resulted in the large variations in cytokine levels reported. Although strict inclusion and exclusion criteria were applied to make the number of studies manageable and the studies itself as comparable as possible, the variety in measurement techniques, biofluid/tissue source and reporting of results complicate comparability. This variety and the lack of adequate control groups in some studies made it impossible to perform a meta-analysis. Nevertheless, this review provides the first comprehensive overview of the current literature on the cytokines IL-17 and IL-23 in human pregnancy, including *in vitro* and animal work. Furthermore, we present summarized graphs of all published mean serum IL-17 levels measured by ELISA in healthy pregnancy and PE pregnancy, and IL-23 levels in PE pregnancy.

## 5 Conclusion

We identified a large body of evidence for an important role of IL-17 in all stages of pregnancy, suggesting that well-controlled balance is required: to establish and maintain pregnancy, an increase in IL-17 is needed, but a too excessive increase is associated with negative consequences. Only minor evidence was found for the role of IL-23 in healthy and complicated pregnancy, due to the low number of studies. The large heterogeneity between and within studies limited further sub analyses. This study provides a pivotal foundation for future research on the interplay between pregnancy, IMIDs, and immunomodulating medication. This research is needed for IMID patients who experience unpredictable disease fluctuations during pregnancy and for whom the inhibition of IL-17 and IL-23 provide great therapeutic efficacy, but reproductive health effects remain unforeseeable.

## CRediT authorship contribution statement

**Aniek Plug:** Writing – original draft, Visualization, Methodology, Formal analysis, Data curation, Conceptualization. **Liana Barenbrug:** Writing – original draft, Visualization, Methodology, Formal analysis, Data curation. **Bart G.J. Moerings:** Writing – review & editing. **Elke M.G. de Jong:** Writing – review & editing. **Renate G. van der Molen:** Writing – review & editing, Supervision, Methodology, Conceptualization.

## Funding

This study was supported by the LEO Foundation (ref. number LF-OC-23-001331). Funders were not involved in the design of the study, the analysis and interpretation of data, writing of the manuscript and the decision to submit the article for publication.

## Declaration of competing interest

The authors declare the following financial interests/personal relationships which may be considered as potential competing interests: Renate G. van der Molen reports financial support was provided by LEO Foundation. Renate G. van der Molen reports a relationship with LEO Foundation that includes: funding grants.Elke M.G.J. de Jong has received research grants for the independent research fund of the Department of Dermatology, 10.13039/501100001832Radboud University Medical Centre (10.13039/501100006209Radboudumc), Nijmegen, The Netherlands, from AbbVie, BMS, Janssen Pharmaceutica, Leo Pharma, Novartis, and UCB for research on psoriasis. She has acted as consultant and/or paid speaker for and/or participated in research sponsored by companies that manufacture drugs used for the treatment of psoriasis or eczema including AbbVie, Amgen, Almirall, Boehringer Ingelheim, BMS, Celgene, Galapagos, Janssen Pharmaceutica, Leo Pharma, Lilly, Novartis, Sanofi and UCB. All funding is not personal but goes directly to the Department of Dermatology, 10.13039/501100001832Radboud University Medical Centre (10.13039/501100006209Radboudumc), Nijmegen, The Netherlands, and is not related to this study. If there are other authors, they declare that they have no known competing financial interests or personal relationships that could have appeared to influence the work reported in this paper.

## Figures and Tables

**Table:** 

**PubMed**	**Embase**	**Web of Science**
Interleukin-17[Mesh] OR Interleukin-23[Mesh:NoExp] OR Interleukin-23 Subunit p19[Mesh] OR "Th17 Cells"[Mesh] OR "17-Helper-T"[tiab] OR CTLA8[tiab] OR CTLA-8[tiab] OR Cytotoxic-T-lymphocyte-Associated-Antigen-8[tiab] OR IL-17∗[tiab] OR IL-17∗[tiab] OR IL-23[tiab] OR IL-23[tiab] OR Interleukin-17∗[tiab] OR Interleukin-23∗[tiab] OR Interleukin-25[tiab] OR "T-Helper-17"[tiab] OR Th17 [tiab] OR TH-17 [tiab]	Interleukin 17/or Interleukin 23/or Interleukin 25/or TH17 cell/(17-Helper-T or CTLA8 or CTLA-8 or Cytotoxic-T-lymphocyte-Associated-Antigen-8 or IL-17∗ or IL-17∗ or IL-23 or IL-23 or Interleukin-17∗ or Interleukin-23∗ or Interleukin-25 or T-Helper-17 or Th17 or TH-17).ti,ab,kf.	TS= (“17-Helper-T” OR CTLA8 OR “CTLA-8” OR “Cytotoxic-T-lymphocyte Associated-Antigen-8” OR IL-17∗ OR “IL-17∗” OR IL-23 OR “IL-23” OR “Interleukin 17∗” OR “Interleukin 23” OR “Interleukin 25” OR “Interleukin-17∗” OR “Interleukin-23∗” OR “Interleukin-25” OR “TH-17” OR Th17 OR “T-Helper-17”)
Decidua[Mesh] OR Maternal Health Services[MeSH:NoExp] OR Placenta[Mesh] OR Preconception Care[MeSH] OR Prenatal Care[Mesh] OR Pregnancy[Mesh] OR Pregnant Women[Mesh] OR Trophoblasts[Mesh] OR Uterus[Mesh:NoExp] OR antenatal∗[tiab] OR ante-natal∗[tiab] OR cytotrophoblast∗[tiab] OR decidu∗[tiab] OR maternal[tiab] OR placenta∗[tiab] OR Preconception∗[tiab] OR Pre-conception∗[tiab] OR pregnanc∗[tiab] OR pregnant[tiab] OR prenatal∗[tiab] OR pre-natal∗[tiab] OR syncytiotrophoblast∗[tiab] OR trophoblast∗[tiab] OR uteri∗[tiab] OR uterus[tiab]	decidua/or exp pregnancy/or exp trophoblast cell line/or fetoplacental unit/or maternal health service/or placenta circulation/or placenta/or pregnant woman/or prenatal care/or prepregnancy care/or trophoblast/or uterus/(antenatal∗ or ante-natal∗ or cytotrophoblast∗ or decidu∗ or maternal or placenta∗ or Preconception∗ or Pre-conception∗ or pregnanc∗ or pregnant or prenatal∗ or pre-natal∗ or syncytiotrophoblast∗ or trophoblast∗ or uteri∗ or uterus).ti,ab,kf.	TS=(decidu∗ OR trophoblast∗ OR fetoplacental OR pregnant OR prepregnancy OR uterus OR antenatal∗ OR “ante-natal∗” OR cytotrophoblast∗ OR maternal OR placenta∗ OR Preconception∗ OR “Pre-conception∗” OR pregnanc∗ OR prenatal∗ OR “pre-natal∗” OR syncytiotrophoblast∗ OR uteri∗ OR uterus)

**Table:** 

**Author**	**Bias due to confounding**	**Bias in selection of participants into the study**	**Bias in classification of interventions**	**Bias due to deviations from intended interventions**	**Bias due to missing data**	**Bias in measurement of outcomes**	**Bias in selection of the reported result**	**Overall risk of Bias**
Allswede, 2020	Low	Low	Low	Low	Low	Low	Low	Low
Anvari, 2015	Low	Moderate	Moderate	Low	Low	Low	Low	Moderate
Barnie, 2015	Moderate	Low	Moderate	Low	Low	Low	Low	Moderate
Batebi, 2019	Low	Low	Low	Low	Low	Low	Low	Low
Bhati, 2023	Moderate	Low	Moderate	Low	Low	Low	Low	Moderate
Brewster, 2008	Low	Low	Low	Low	Low	Low	Moderate	Moderate
Buttrup Larsen, 2018	Moderate	Low	Low	Low	Moderate	Low	Moderate	Moderate
Buxton, 2018	Moderate	Low	Moderate	Low	Low	Low	Low	Moderate
Cai, 2016	Moderate	Low	Moderate	Low	Low	Low	Low	Moderate
Cao, 2015	Moderate	Low	Low	Low	Low	Low	Moderate	Moderate
Charkiewicz, 2018	Low	Low	Low	Low	Low	Low	Low	Low
Chaiworapongsa, 2024	Low	Low	Low	Low	Low	Low	Moderate	Moderate
Chen, 2019	Moderate	Low	Low	Low	Low	Low	Low	Moderate
Chen, 2022	Moderate	Low	Low	Low	Moderate	Low	Low	Moderate
Chow, 2008	Low	Low	Low	Low	Low	Low	Low	Low
Darmochwal, 2017	Low	Low	Low	Low	Low	Low	Moderate	Moderate
Darmochwal, 2022	Low	Low	Low	Low	Low	Low	Moderate	Moderate
Ding, 2023	Low	Low	Low	Low	Low	Low	Low	Low
Eghbal-Fard, 2019	Moderate	Low	Low	Low	Low	Low	Low	Moderate
El Shahaway, 2019	Low	Low	Low	Low	Low	Low	Low	Low
Forghani, 2023	Low	Low	Low	Low	Low	Low	Low	Low
Gharesi Fard, 2019	Low	Low	Low	Low	Low	Low	Low	Low
Giannubilo, 2012	Moderate	Low	Moderate	Low	Low	Low	Low	Moderate
Hao, 2015	Low	Low	Low	Low	Low	Low	Low	Low
Hee, 2011	Low	Moderate	Serious	Low	Moderate	Low	Low	Serious
Holtan, 2015	Moderate	Low	Moderate	Low	Low	Low	Low	Moderate
Jahromi, 2014	Low	Low	Low	Low	Low	Low	Low	Low
Jonsson, 2006	Moderate	Moderate	Moderate	Low	Moderate	Low	Low	Moderate
Kaur, 2021	Low	Low	Low	Low	Low	Low	Low	Low
Khalaf, 2021	Low	Low	Low	Low	Low	Low	Low	Low
Kieffer, 2020	Low	Low	Low	Low	Low	Low	Moderate	Moderate
Kieffer 2019	Low	Low	Low	Low	Low	Low	Low	Low
Ku, 2023	Low	Low	Low	Low	Low	Low	Low	Low
Lang 2021	Low	Low	Low	Low	Low	Low	Low	Low
Li 2017	Moderate	Low	Moderate	Low	Low	Low	Low	Moderate
Liu, 2023	Low	Low	Low	Low	Low	Low	Low	Low
Marcoli et al.,. 2015	Moderate	Moderate	Serious	Low	Low	Low	Low	Serious
Martinez-Garcia, 2011	Moderate	Moderate	Serious	Low	Low	Low	Low	Serious
Molvarec, 2015	Low	Low	Low	Low	Low	Low	Low	Low
Mundim, 2016	Low	Low	Low	Low	Low	Low	Low	Low
Nakashima, 2010	Moderate	low	Moderate	Low	Low	Low	Low	Moderate
O'Hern Perfetto 2015	Low	Low	Low	Low	Low	Low	Low	Low
Ozkan 2013	Low	Low	Low	Low	Low	Low	Low	Low
Perucci, 2023	Moderate	Low	Low	Low	Low	Low	Low	Moderate
Pongcharoen, 2007	Moderate	Moderate	Serious	Low	Low	Low	Low	Serious
Poordast, 2017	Low	Low	Low	Low	Low	Low	Low	Low
Qian, 2018	Low	Low	Low	Low	Low	Low	Low	Low
Ribeiro, 2017	Low	Low	Low	Low	Low	Low	Moderate	Moderate
Salazar Garcia, 2018	Low	Low	Low	Low	Moderate	Low	Low	Moderate
Shafiq 2021	Low	Low	Low	Low	Low	Low	Low	Low
Shen, 2023	Low	Low	Low	Low	Low	Low	Low	Low
Son, 2015	Low	Low	Low	Low	Low	Low	Low	Low
Tagoma, 2019	Low	Low	Low	Low	Low	Low	Low	Low
Tangeras, 2015	Low	Low	Low	Low	Low	Low	Low	Low
Uz, 2010	Moderate	Moderate	Serious	Low	Low	Low	Low	Serious
Walsh, 2021	Moderate	Low	Moderate	Low	Low	Low	Low	Moderate
Wang, 2021	Low	Low	Low	Low	Moderate	Low	Moderate	Moderate
Weissenbacher, 2012	Low	Low	Low	Low	Moderate	Low	Low	Moderate
Wu, 2014	Moderate	Moderate	Serious	Low	Low	Low	Low	Serious
Yu, 2017	Low	Low	Low	Low	Low	Low	Moderate	Moderate
Zhang, 2017	Low	Low	Low	Low	Low	Low	Low	Low
Zhao, 2022	Moderate	Low	Low	Low	Low	Low	Low	Moderate
Zhu, 2017	Moderate	Low	Low	Low	Low	Low	Low	Moderate
Zigansa, 2012	Moderate	Low	Moderate	Low	Low	Low	Low	Moderate

**Table:** 

Author	Quality of evidence	
Allswede, 2020	2	Prospective comparative cohort trial
Anvari, 2015	4	Cross-sectional study
Barnie, 2015	4	Cross-sectional study
Batebi, 2019	4	Cross-sectional study
Bhati, 2023	4	Cross-sectional study
Brewster, 2008	4	Cross-sectional study
Buttrup Larsen, 2018	4	Cross-sectional study
Buxton, 2018	3	Retrospective cohort study
Cai, 2016	4	Cross-sectional study
Cao, 2015	4	Cross-sectional study
Charkiewicz, 2018	4	Cross-sectional study
Chaiworapongsa, 2024	3	Case-control study
Chen, 2019	4	Cross-sectional study
Chen, 2022	2	Prospective comparative cohort trial
Chow, 2008	4	Cross-sectional study
Darmochwal, 2017	4	Cross-sectional study
Darmochwal, 2022	4	Cross-sectional study
Ding, 2023	4	Cross-sectional study
Eghbal-Fard, 2019	4	Cross-sectional study
El Shahaway, 2019	2	Prospective comparative cohort trial
Forghani, 2023	4	Cross-sectional study
Gharesi Fard, 2019	4	Cross-sectional study
Giannubilo, 2012	4	Cross-sectional study
Hao, 2015	4	Cross-sectional study
Hee, 2011	3	Case-control study & retrospective cohort study
Holtan, 2015	3	Retrospective cohort study
Jahromi, 2014	4	Cross-sectional study
Jonsson, 2006	4	Cross-sectional study
Kaminski, 2018	4	Cross-sectional study
Kaur, 2021	4	Cross-sectional study
Khalaf, 2021	2	prospective comparative cohort trial
Kieffer, 2020	4	Cross-sectional study
Kieffer 2019	4	Cross-sectional study
Ku, 2023	2	Prospective comparative cohort trial
Lang 2021	4	Cross-sectional study
Li 2017	4	Cross-sectional study
Liu, 2023	4	Cross-sectional study
Martinez-Garcia, 2011	2	Prospective comparative cohort trial
Molvarec, 2015	4	Cross-sectional study
Nakashima, 2010	4	Cross-sectional study
O'Hern Perfetto 2015	4	Cross-sectional study
Ozkan 2013	4	Cross-sectional study
Perucci, 2023	4	Cross-sectional study
Pongcharoen, 2007	4	Cross-sectional study
Poordast, 2017	4	Cross-sectional study
Qian, 2018	4	Cross-sectional study
Ribeiro, 2017	4	Cross-sectional study
Salazar Garcia, 2018	3	Case-control study
Shafiq 2021	2	Prospective comparative cohort trial
Shen, 2023	3	Case-control study
Son, 2015	3	Case-control study
Tagoma, 2019	4	Cross-sectional study
Tangeras, 2015	3	Case-control study
Uz, 2010	4	Cross-sectional study
Walsh, 2021	4	Cross-sectional study
Wang, 2021	4	Cross-sectional study
Weissenbacher, 2012	4	Cross-sectional study
Yu, 2017	4	Cross-sectional study
Zhang, 2017	4	Cross-sectional study
Zhao, 2022	3	Case-control study
Zhu, 2017	4	Cross-sectional study
Zigansa, 2012	2	Prospective comparative cohort trial

**Table:** 

**Study**	**Cell/tissue type**	**Treatment**	**Dose**	**Exposure time**	**Outcome measure**	**Findings**
IL-17 treatment						
Wu et al., 2014 [20]	Primary first trimester trophoblasts	Recombinant IL-17A	*Proliferation:* 1, 10, 50, 200, 400 ng/ml *Invasion:* 0, 5, 10, 50, 100, 200 ng/ml *Apoptosis:* 50 ng/ml	48 h	Proliferation (BrdU assay) Invasion (invasion assay) Apoptosis (Annexin V/PI cell apoptosis assay)	Dose-dependent increased proliferation Increased invasion (10 ng/ml: P = 0.008, 50 ng/ml: P = 0.001, 100 ng/ml: P = 0.000, 200 ng/ml: P = 0.000) No effect on apoptosis (P = 0.066)
Pongcharoen et al., 2006 [21]	JEG-3	Recombinant IL-17	1, 10, 100 ng/ml	48 h	Proliferation (BrdU assay) Invasion (transwell invasion assay)	No effect on trophoblast proliferation. Increased invasion (72 h: P = 0.03, P = 0.007, P = 0.01 for 1, 10 and 100 ng/ml, resp. 96 h: P = 0.0001, P = 0.0004, P = 0.001 for 1, 10 and 100 ng/ml, resp.)
You et al., 2021 [22]	Swan 71 (3D BLS)	Recombinant IL-17	200 ng/ml	24 h	Migration (BLS migration assay)	Increased attachment and migration (at 48h: P < 0.05, at 72h: P < 0.01)
Liu et al., 2021 [23]	HTR-8/SVneo	Recombinant IL-25 and/or anti-IL-25 neutralizing antibody	*Cell proliferation:* *IL-25:* 0, 12.5, 25, 50, 100, 200 ng/ml *IL-25:* 50 ng/ml + *Anti-IL-25 :* 1 μg/ml. *Invasion:* *IL-25:* 50 ng/ml *Anti-IL-25:* 1 μg/ml	24 h	Proliferation (CCK-8) Invasion (matrigel-coated transwell chamber)	No effect on apoptosis (P = 0.066) Increased invasion for IL-25 vs. control and vs. IL-25 + anti-IL-25 (P < 0.05)
Zhang et al. 2022 [24]	HTR-8/SVneo	IL-17 overexpressing plasmids or siRNA-IL-17	*Not specified*	48 h	Viability/proliferation (CCK-8/colony formation assay, resp.) Migration (transwell migration assay) Invasion (transwell migration assay) MMP-3, MMP-2/9 and Vimentin mRNA and protein expression (RT-PCR and western blot)	IL-17 increased proliferation (P < 0.01). siRNA-IL-17 decreased proliferation (P < 0.01) IL-17 increased cell viability (P < 0.01). siRNA-IL-17 decreased cell viability. IL-17 increased migration (P < 0.01). siRNA-IL-17 decreased migration (P < 0.01). IL-17 increased invasion (P < 0.01) siRNA-IL-17 decreased invasion (P < 0.01). IL-17 increased MMP-3, MMP-2/9 and vimentin mRNA (P < 0.01) and protein expression. siRNA-IL-17 decreased MMP-3, MMP-9 and vimentin mRNA (P < 0.01) and protein expression.
IL-17 production						
Marcoli et al., 2015 [25]	Human early and term placental explants	NA	NA	For 2 weeks every 3 days supernatant was collected and pooled for analyses.	IL-17 expression in placental explant supernatant (Bioplex assay)	Early placental explants: 153.5 pg/ml Term placental explants: 29.8 pg/ml
Ostojic et al., 2003 [26]	Mouse placental and decidual explants (abortion prone pregnancy model CBA⁄J·DBA⁄2 and CBA/J-BALB/c non abortion prone model)	NA	NA	*Not specified*	IL-17 expression in placental explant supernatant (ELISA)	Abortion prone placental explants: 1.28 pg/ml Non-abortion prone placental explants: 1.36 pg/ml Abortion prone decidual explants: 1.66 pg/ml Non-abortion prone decidual explants: 1.22 pg/ml NS
IL-23 treatment						
He et al., 2022 [27]	HTR-8/SVneo	Recombinant IL-23	50, 100 ng/ml	*Not specified*	Proliferation (CCK-8) Migration (wound healing assay) Invasion (transwell assay) MMP2, MMP9, E-cadherin and N-cadherin protein expression (western blot) Apoptosis (TUNEL assay)	Decreased proliferation (100 ng/ml at 72h and 96h: P < 0.001, 50 ng/ml at 96h: P < 0.5). Decreased migration (50 ng/ml: P < 0.001, 100 ng/ml: P < 0.001) Decreased invasion (50 ng/ml: P < 0.5, 100 ng/ml: P < 0.001) Increased E-cadherin and decreased MMP2, MMP9 and N-cadherin expression Increased apoptosis (50 ng/ml: P < 0.01, 100 ng/ml: P < 0.001)

BrdU: Bromodeoxyuridine. BLS: blastocyst-like spheroids. CCK-8: cell counting kit-8. siRNA: small-interfering RNA. MMP: matrix metalloproteinases. RT-PCR: reverse transcription polymerase chain reaction. NA: not applicable. ELISA: enzyme-linked immunosorbent assay.

**Table:** 

Study	Animal (species)	Model	Population size	Intervention	Outcome measures (technique)	Gestational age (days) at measurement	Findings
IL-17							
Ostojic et al., 2003 [26]	Mouse (CBA/J-BALB/c)	Normal pregnancy	3	NA	Placental and decidual IL-17 expression (IHC)	8.5, 9.5 and 12.5	No expression of IL-17 in placenta. Low IL-17 expression in stroma and glandular epithelium of the decidua.
Lewis et al., 2018 [31]	Mouse (C57BL/6J x congenic B6.SJL-PtprcaPepcb /BoyJ)	Normal pregnancy	6	NA	Cytokines in placental protein lysate (ELISA)	15 and 19	(#*Mean ± SE)* Placental IL-17at 15 days*:* 0.0017 ± 0.0002 pg/ug protein Placental IL-17at 19 days: 0.0017 ± 0.0002 pg/ug protein. NS
Orsi et al., 2007 [32]	Mouse (CD-1)	Normal pregnancy	Mid pregnancy: 15 Late pregnancy: 18	NA	Serum and amniotic fluid IL-17 (Multiplex immunoassay)	11 and 18	(#*Mean ± #SEM)*: Serum IL-17 day 11 126.0 *±* 44.5 pg/ml Serum IL-17 day 18*:* 882.4 ± 101.2 pg/ml Serum IL-17 day 11 versus day 18: P < 0.001 Amniotic fluid IL-17 day 11 versus day 18∗ NS
Travis et al., 2019 [28]	Rat (Sprague Dawley)	Normal pregnancy	Infused: 8 Control: 8	Chronic recombinant IL-17A/F infusion. 100 pg/day GA: 12–19 days	NK-cell number in circulation and placenta Placental NK-cell activity Placental inflammatory cytokines, cytolytic proteins and ROS Circulating cytokines and cytolytic proteins MAP Renal ROS Uterine artery endothelial function Fetal and placental weight	19	Increased total and cytolytic NK-cell number in the circulation and placenta Increased placental NK-cell activity Increased IL-12, IFN-γ, MIP3-α, granzyme A, B and K, and ROS Increased circulating cytolytic proteins granzyme B and K Decreased fetal and placental weight Increased MAP Increased renal ROS Decreased vascular endothelial function Decreased fetal and placental weight Important: although infusion was successful, placental IL-17 was not changed
Travis et al., 2020 [29]	Rat (Sprague Dawley)	RUPP (PE)	RUPP: 12 Control: 12	Chronic IL-17 receptor C infusion. 100 pg/day GA: 14–19 days	Th17 number Maternal MAP Fetal weight, litter size, live pups, placental weight Circulating and placental NK-cell number and cytolytic activity Placental oxidative stress Placental and circulating cytokines and cytolytic proteins Placental and circulating angiogenic factors	19	Increased Th17 cell number Decreased maternal MAP Increased fetal and placental weight Increased number of live pups Decreased placental total and cytolytic NK-cell number Decreased placental oxidative stress Decreased placental IL-6, IL-12, TNF-α, and MIP-3α Decreased circulating IL-6, IL-12, TNF-α and IFN-γ Increased VEGF and decreased s-Flt1
Wallace et al., 2014 [30]	Rat (Sprague-Dawley)	HELLP	HELLP: 10 Control: 10	sFlt-1(4.7 mg/kg/day) and sEndoglin (7 mg/kg/day), infusion to induce HELLP model. GA: 12–19 days	Plasma IL-17 (ELISA)	Plasma IL-17 (ELISA)	(#*Mean ± #SEM)* Plasma IL-17 HELLP 7.3 ± 1.7 pg/ml Plasma IL-17 normal pregnancy 3.0 ± 0.7 pg/ml p = 0.04
Xu et al., 2016 [33]	Mouse (CBA/J x BALB/c (*normal pregnancy*) and CBA/J x DBA/2 (*abortion prone*))	Abortion	Abortion: 10 Control: 10	Recombinant IL-17 injection 200ul of 10ug/ml GA: 4 Anti-IL-17 injection 200 μl of 20 μg/ml GA: 4, 6, 8 days. MIA	Abortion rate Splenic Treg and Th17 cell frequencies (flowcytometry) Serum IL-17 protein (ELISA) Placental IL-17, RORγT and Foxp3 mRNA (RT-PCR) and protein (western blot)	14	Normal pregnancy: IL-17 induced fetal loss, increased splenic Th17, decreased splenic Treg, increased placental IL-17, RORγT and Foxp3 mRNA and IL-17 and RORγT protein expression Abortion prone pregnancy: anti-IL17 prevented fetal loss, decreased splenic Th17, increased splenic Treg, decreased serum IL-17, decreased placental IL-17 and RORγT mRNA and protein and increased Foxp3 mRNA and protein
Li et al., 2018 [34]	Mouse (CBA/J x BALB/c (*normal pregnancy*) and CBA/J x DBA/2 (*abortion prone*))	Abortion	Abortion: 10 Control: 10	NA	Serum and decidual IL-17A (ELISA)	14	(#*Mean ± #SD)* Serum IL-17A normal pregnancy model: 14.0 ± 2.2 pg/ml Serum IL-17A abortion model: 27.5 ± 1.5 pg/ml Decidua IL-17A normal pregnancy model: 89.3 ± 12.0 pg/ml Decidua IL-17A abortion model: 157.4 ± 17.0 pg/ml
Pang et al., 2019 [35]	Mouse (C57BL/6 x BALB/c)	MIA	MIA: 24 Control: 24	Intraperitoneal LPS injection. 2.5 μg/20 g-mice. GA: 8.5 days	Serum IL-17A (ELISA)	10.5	(#*Mean ± SEM):* Increased serum IL-17A: 328.5 ± 17.3 pg/ml vs. 188.9 ± 10.5 pg/ml)
Garcia-Valtanen et al., 2019 [36]	Mouse (C57BI/6J)	MIA	MIA: 5 Control: 5	Poly (I:C) injection, 20 mg/kg GA: 12 days	Serum IL-17A (Bead-based immune assay)	12 and 14	(#*Mean ± SEM)* Serum IL-17 MIA: 16.8 ± 7.2 pg/ml. Serum IL-17 control: 7.0 ± 0.9 pg/ml NS
IL-23							
Mas et al., 2008 [37]	Mouse (Swiss)	Normal pregnancy	3	NA	Placental IL-23 expression (IHC)	10	Only weak IL-23 expression in spongiotrophoblast at 10 weeks GA.
Lewis et al., 2018 [31]	Mouse (C57BL/6J x congenic B6.SJL-PtprcaPepcb/BoyJ	Normal pregnancy	6	NA	Cytokines in placental protein lysate (ELISA)	15 and 19	(#*Mean ± SE)* Placental IL-23 at 15 days*:* 0.017 ± 0.005 pg/ug protein Placental IL-23 at 19 days: 0.021 ± 0.005 pg/ug protein. NS

NA: not applicable. IHC: immunohistochemistry. ELISA: enzyme-linked immunosorbent assay. SE: standard error. NS: not significant. SEM: standard error of the mean. GA: gestational age. NK: natural killer. ROS: reactive oxygen species. MAP: mean arterial pressure. IFN-γ: interferon-γ. MIP-3α: macrophage inflammatory protein-3α. RUPP: reduced uterine perfusion pressure. Th17: T-helper 17. TNF-α: tumor necrosis factor-α, Treg: regulatory T-cells. VEGF: vascular endothelial growth factor. S-Flt1: soluble fms related receptor tyrosine kinase 1. HELPP: hemolysis, elevated liver enzymes, and low platelets. RORγT: RAR-related orphan receptor-γ. Foxp3: forkhead box P3. RT-PCR: Reverse transcription polymerase chain reaction. MIA: maternal immune activation. LPS: lipopolysaccharide. Poly(I:C): Polyinosinic:polycytidylic acid.

#: values extracted with WebPlotDigitizer.

∗Amniotic fluid IL-17 concentrations were too low to be extracted with the WebPlotDigitizer.

**Table:** 

**Study**	**Location**	**Case population size**	**Control population size**	**Outcome measures (technique)**	**Gestational age at measurement of cases**	**Gestational age at measurement of controls**	**IL-17 level cases group**	**IL-17 level control group**	**P-value**
**Healthy pregnancy (cases): vs. non-pregnancy (controls):**									
Pongcharoen et al., 2007 [53]	Thailand	5	NA	IHC and RT-PCR	Term	NA	Weak to moderate IL-17 expression in cytotrophoblasts. Moderate to strong IL-17 expression in syncytiotrophoblasts. IL-17 mRNA expression in term placenta	NA	NA
Holtan et al., 2015 [38]	USA	16	11	Plasma IL-17 (Milliplex assay)	18 samples during pregnancy. Baseline sample in first trimester (average 9.7 weeks)	NA	Median (range) 5.5 (∗-46.9) pg/ml	Median (range) 14.8 (2.2–161.6) pg/ml	P = 0.03 An unconditional growth model did not show significant change of IL-17 levels during pregnancy.
Khalaf et al., 2021 [39]	Egypt	40	90	Serum IL-17A (ELISA)	Between 7 and 10 weeks	NA	#Mean ± SEM 21.3 ± 2.0 pg/ml	#Mean ± SEM 21.1 ± 2.0 pg/ml	NS
Jahromi et al., 2014 [40]	Iran	Total: 97 First trimester: 32 Second trimester: 25 Third trimester 40	28	Serum IL-17 (ELISA)	Mean ± SD Total: 25.81 ± 9.9 weeks First trimester: 11.2 ± 1.6 weeks Second trimester: 22.2 ± 2.7 weeks Third trimester: 32.9 ± 4.3 weeks	NA	Mean ± SD Total: 24.96 ± 15.75 pg/ml First trimester: 9.52 ± 6.21 pg/ml Second trimester: 20.24 ± 6.75 pg/ml Third trimester: 40.25 ± 10.10 pg/ml	Mean ± SD 2.59 ± 2.05 pg/ml	Total: P < 0.001 First, second and third trimester separately vs control: all P < 0.01 Second vs. first trimester: P < 0.001 Third vs. first trimester: P < 0.001
Perucci et al., 2023 [41]	Brazil	34	32	Plasma IL-17A (ELISA)	Median (25th–75th percentiles) 31 (30–35) weeks	NA	Mean ± SD 0.95 ± 0.26 ng/ml	Mean ± SD 0.41 ± 0.13 ng/ml	P < 0.001
Shen et al., 2023 [42]	China	18	30	Serum IL-17A and IL-17F (multiplex immunoassay)	Mean ± SD 40.77 ± 2.575 weeks (within 24 h before delivery; vaginal delivery/total = 6/18)	NA	Median (IQR) IL-17A: 9.61 (5.94–14.33) pg/ml IL-17F: 4.35 (4.01–5.37) pg/ml	Median (IQR) IL-17A: 5.68 (4.59–6.69) pg/ml IL-17F: 3.86 (3.79–3.91) pg/ml	IL-17A: P = 0.010 IL-17F: P = 0.003
Tagoma et al., 2019 [43]	Estonia	115	42	Plasma IL-17A and IL-17F (multiplex magnetic bead assay)	Mean ± SD 26.2 ± 1.6 weeks	NA	Median (25‐75th percentiles) IL-17A: 8.9 (4.6–15.3) pg/ml IL-17F: 15.1 (0.0–46.6) pg/ml	Median (25‐75th percentiles) IL-17A: 4.5 (0.8–11.5) pg/ml IL-17F: 98.5 (53.7–144.1) pg/ml	Not provided
Lang et al., 2021 [44]	China	102	147	Serum IL-17A (ELISA)	Third trimester	NA	Mean ± SEM 61.13 ± 1.43 pg/ml	Mean ± SEM 59.1 ± 0.93 pg/ml	Not provided
Poordast et al., 2017 [45]	Iran	30	30	Serum IL-17 (ELISA)	Third trimester	NA	#Mean ± SEM 382 ± 27 pg/ml	#Mean ± SEM 336 ± 25 pg/ml	NS
Chen et al., 2019 [46]	China	27	10	Plasma IL-17 (ELISA)	Mean ± SD 37 ± 2.1 (at term, CS delivery)	NA	#Mean ± SD 36.2 ± 27.7 pg/ml	#Mean ± SD 52.8 ± 17.4 pg/ml	NS
Longitudinal measurement of healthy pregnancy									
Allswede et al., 2020 [47]	US	442	NA	Serum IL-17A (multiplex bead assay)	Between 7 and 48 weeks at each prenatal visit (2-3x, total of 1214 samples)	NA	Not provided	NA	Not provided "The cytokine pattern was best described by linear trend showing decreasing IL-17A concentration across gestation"
Darmochwal-Kolarz et al., 2022 [48]	Poland	45	NA	Serum IL-17A (ELISA)	Mean ± SD First trimester: 10 ± 2 weeks Second trimester: 20 ± 2 weeks Third trimester 32 ± 2 weeks	NA	Mean ± SD First trimester: 1.12 ± 0.42 pg/ml Second trimester: 1.63 ± 0.56 pg/ml Third trimester: 2.35 ± 0.53 pg/ml	NA	First vs. second trimester: P < 0.05 Second vs. third trimester P < 0.05
Martínez-García et al., 2011 [49]	Mexico	13	NA	Serum IL-17 (ELISA)	First trimester (10–12 weeks) Second (24–26 weeks) Third trimester (36–38 weeks)	NA	Mean (range) First trimester: 14.61 (0–59.81) pg/ml Second trimester 21.4 (0–62.79) pg/ml Third trimester 37.28 (0–62.79) pg/ml	NA	First vs. third trimester: P < 0.05 ANOVA: P = 0.0198
Hee et al., 2011 [50]	Denmark	40	NA	Serum IL-17A (multiplex sandwich immunoassay)	12, 19, 26, 33 and 39 weeks (for week 39: n = 21)	NA	Median (IQR) 12 weeks: 291 (66–593) pg/ml 19 weeks: 12 (<4–778) pg/ml 26 weeks: 53 (134–461) pg/ml 33 weeks: 198 (115–524) pg/ml 39 weeks: 180 (143–259) pg/ml	NA	Overall: P = 0.006 Pairwise comparisons: P = 0.33–0.91
Chow et al., 2008 [51]	Australia	100 (elective amniocentesis due to older maternal age (range: 30–47 years, median: 37 years)	NA	Amniotic fluid (n = 100) and serum (n = 33) IL-17 (multiplex bead-based immunoassay)	14–16 weeks	NA	Median (range) Serum: 6.28 (0.59–106.60) pg/ml, detectable levels in 88 % of samples Amniotic fluid: 4.17 (0.47–20.13) pg/ml, detectable levels in 15 % of samples	NA	Serum vs. amniotic fluid: P = 0.02
Weissenbacher et al., 2012 [52]	Germany	66	NA	Amniotic fluid IL-17 (ELISA)	Median (range) Mid-trimester: 17 (15–31) weeks At CS: 39 (30–42) weeks	NA	Median (range) Mid-trimester: 17.5 (4.6–42.4) pg/ml At CS: 23.1 (3.9–46.0) pg/ml	NA	Mid-trimester vs. at CS: P = 0.645
**Pregnancy complications**									
**PE pregnancy (cases). vs. healthy pregnancy (controls)**									
Liu et al., 2023 [54]	China	34	34	Serum IL-17 (ELISA)	11–13 weeks	11–13 weeks	Median (IQR) 3.69 (3.10, 4.44) pg/ml	Median (IQR) 3.51 (3.10–4.29) pg/ml	P = 0.681
Salazar Garcia et al., 2018 [55]	US	8	77	Serum IL-17A (multiplex bead-based assay)	Mean ± SD 11.6 ± 2.7 weeks	Mean ± SD 10.4 ± 2.6 weeks	Median (25–75 percentiles) 5.2 (3.9–10.4) pg/ml	Median (25–75 percentiles) 4.3 (3.0−10.4) pg/ml	NS
Chen et al., 2022 [56]	Taiwan	39	127	Plasma IL-17A (ELISA)	14–27 weeks (second trimester) 28–39 weeks (third trimester)	14–27 weeks (second trimester) 28–39 weeks (third trimester)	Median (IQR) Second trimester: 3.29 (1.44, 5.37) pg/ml Third trimester: 3.29 (1.92, 5.97) pg/ml	Median (IQR) Second trimester: 2.01 (1.46, 3.22) pg/ml Third trimester: 2.52 (1.48, 3.54) pg/ml	Second trimester: P = 0.02 Third trimester: P = 0.02
Walsh et al., 2022 [57]	US	45	36	Plasma IL-17A (ELISA)	13–28 weeks (second trimester) 29–40 weeks (third trimester)	13–28 weeks (second trimester) 29–40 weeks (third trimester)	#? (95 % CI) Second trimester: 7.24 (0.783–20.3) pg/ml Third trimester: 7.24 (1.16–15.1) pg/ml	#? (95 % CI) Second trimester: 4.17 (0–13.8) pg/ml Third trimester: 6.36 (0.909–12.7) pg/ml	Second trimester: P < 0.01 Third trimester: NS
Shen et al., 2023 [42]	China	19	18	Serum IL-17A and IL-17F (multiplex immunoassay)	Mean ± SD 37.69 ± 4.248 weeks (within 24 h before delivery; vaginal delivery/total = 12/19)	Mean ± SD 40.77 ± 2.575 weeks (within 24 h before delivery; vaginal delivery/total = 6/18)	Median (IQR) IL-17A: 6.80 (3.09–13.02) pg/ml IL-17F: 4.54 (1.91–9.73) pg/ml	Median (IQR) IL-17A: 9.61 (5.94–14.33) pg/ml IL-17F: 4.35 (4.01–5.37) pg/ml	IL-17A: P = 0.213 IL-17F: P = 0.988
Lang et al., 2021 [44]	China	115	102	Serum IL-17A (ELISA)	Third trimester	Third trimester	Mean ± SEM 746.7 ± 17.16 pg/ml	Mean ± SEM 61.13 ± 1.43 pg/ml	P < 0.0001
Poordast et al., 2017 [45]	Iran	30	30	Serum IL-17 (ELISA)	Third trimester	Third trimester	#Mean ± SEM 515 ± 23 pg/ml	#Mean ± SEM 382 ± 27 pg/ml	P < 0.0001
Darmochwal-Kolarz et al., 2022 [48]	Poland	35	45	Serum IL-17A (ELISA)	Mean ± SD 32 ± 3 weeks	Mean ± SD 32 ± 2 weeks	Mean ± SD 3.90 ± 1.35 pg/ml	Mean ± SD 2.35 ± 0.53 pg/ml	P < 0.01
Anvari et al., 2015 [58]	Iran	Peripheral serum: 40 Placentas: 19	Peripheral serum: 40 Placentas: 19	Peripheral serum and placental blood IL-17A (ELISA) Fetal and maternal placental IL-17A expression (RT-qPCR)	At term, CS delivery	At term, CS delivery	Mean ± SD Peripheral serum: 12 ± 6.7 pg/ml Placental blood: 48.7 ± 36.2 pg/ml Mean ± SD (=ΔCt) Maternal placenta: 22.3 ± 0.61 a.u. Fetal placenta: 20.49 ± 0.76 a.u.	Mean ± SD Peripheral serum: 14.5 ± 7.4 pg/ml Placental blood: 23.2 ± 14.3 pg/ml Mean ± SD (=ΔCt) Maternal placenta: 22.5 ± 0.50 a.u. Fetal placenta: 20.5 ± 0.44 a.u.	Peripheral serum: P = 0.14 Placental blood: P < 0.009 Overall PE vs. controls: NS Fetal vs. maternal placenta PE: P < 0.000 Fetal vs. maternal placenta controls: P < 0.000
Tangeras et al., 2015 [59]	Norway	25	504	Serum IL-17A (Multiplex immunoassay)	Median (IQR) 13 (13-13) weeks	Median (IQR) 13 (12–13) weeks	Median (IQR) 95.4 (79.0–120.1) pg/ml	Median (IQR) 97.9 (80.2–123.0) pg/ml	NS
Barnie et al., 2015 [60]	China	17	17	Serum IL-17 (ELISA)	Before delivery	Before delivery	#Mean ± SEM 3.80 ± 0.70 pg/ml	#Mean ± SEM 1.28 ± 0.47 pg/ml	P < 0.05
Batebi et al., 2019 [61]	Iran	Total: 100 Mild PE: 46 Severe PE: 54	100	Serum IL-17A (ELISA)	Not provided	Not provided	Mean ± ? Total: 2.79 ± 1.26 pg/ml Mild PE: 2.5 ± 1.29 pg/ml Severe PE: 3 ± 1.2 pg/ml	Mean ± ? 2.86 ± 1.69 pg/ml	Total PE vs. control: P = 0.73 Mild PE vs. control: P = 0.14 Severe PE vs control: P = 0.32 Mild vs. severe PE: P = 0.46
Brewster et al., 2008 [62]	UK	EO-PE: 7 LO-PE: 13	Early: 7 Late: 13	Serum IL-17 (fluid-phase multiplex immunoassay)	EO-PE: 32 (26–34) weeks LO-PE: 37 (35–40) weeks	Early: 27 (26–30) Late: 39 (35–41)	Mean ± SEM EO-PE: 2.63 ± 1.87 pg/ml LO-PE: 0.23 ± 0.23 pg/ml	Mean ± SEM Early: 2.45 ± 1.55 pg/ml Late: 0.94 ± 0.69 pg/ml	EO-PE vs. early control: NS LO-PE vs. late control: NS
Buttrup Larsen et al., 2018 [63]	Denmark	GH/moderate PE: 9 Severe PE/HELLP: 32	10	Serum IL-17A (ELISA)	Median (IQR) First trimester: exact GA not provided Third trimester GH/moderate PE: 34 (32–38) weeks Third trimester severe PE/HELLP: 36 (33–38) weeks	Median (IQR) First trimester: exact GA not provided Third trimester: 35 (30–37) weeks	#Mean ± SEM First trimester GH/moderate PE: 0.203 ± 0.207 ng/l First trimester severe PE/HELLP: 3.32 ± 1.41 ng/l Third trimester GH/moderate PE: 0.513 ± 0.280 ng/l Third trimester severe PE/HELLP: 1.77 ± 0.62 ng/l	#Mean ± SEM First trimester: 0.272 ± 0.242 ng/l Third trimester: 0.0993 ± 0.0806 ng/l	NS (unclear which comparisons were made)
Cao et al., 2015 [64]	China	Mild PE: 22 Severe PE: 20	22	Serum IL-17A (ELISA)	Mean ± SD Mild PE: 36.42 ± 1.62 weeks Severe PE: 36.62 ± 1.57 weeks	Mean ± SD 38.57 ± 0.98 weeks	#Mean ± SD Mild PE: 1.50 ± 0.09 pg/ml Severe PE: 1.64 ± 0.04 pg/ml	#Mean ± SD 1.09 ± 0.16 pg/ml	Mild PE vs. control: P = 0.003 Severe PE vs. control: P = 0.006
Darmochwal-Kolarz et al., 2017 [65]	Poland	34 (PE-associated FGR)	35	Serum IL-17A (ELISA)	32.05 ± 2.14 weeks	32.62 ± 1.63 weeks	Median (IQR) 3.9 (2. 55–5.06) pg/ml	Median (IQR) 2.4 (1.78–3.11) pg/ml	P < 0.01
Eghbal-Fard et al., 2019 [66]	Iran	50	50	Serum IL-17 (ELISA)	Mean ± SD 35.8 ± 5.0 weeks	Mean ± SD 37.1 ± 2.3 weeks	Mean ± SD 20.80 ± 9.151 pg/ml	Mean ± SD 16.45 ± 4.536 pg/ml	P = 0.064
El Shahaway et al., 2019 [67]	Egypt	20	20	Serum IL-17A (ELISA)	Mean ± SD 33.6 ± 1.7 weeks Median (range) 34 (30–36) weeks	Mean ± SD 33 ± 2.6 weeks Median (range) 34 (28–36) weeks	Mean ± SD 18.5 ± 4.5 pg/ml Median (range) 18.8 (11.2–25) pg/ml	Mean ± SD 4.38 ± 3.6 pg/ml Median (range) 5 (0.4−0) pg/ml	P = 0.001
Jonsson et al., 2006 [68]	Sweden	15	15	Serum IL-17 (multiplex immunoassay)	Mean (range) 34 (29–40) weeks	Mean (range) 33 (29–37) weeks	Median (range) 0.0 (0.0–0.0)pg/ml	Median (range) 0.0 (0.0–3.6) pg/ml	P = 0.528
Molvarec et al., 2015 [69]	Hungary	59	60	Serum IL-17A (ELISA)	Median (IQR) 37 (36–39) weeks	Median (IQR) 36 (36–37) weeks	Median (IQR) 0.47 (0–0.53) pg/ml (Measurable serum IL-17 level in 54.2 %)	Median (IQR) 0 (0-0) pg/ml (Measurable serum IL-17 level in 5.0 %)	P < 0.001
Wang et al.,. 2021 [70]	China	PE: 60 Mild PE: 33 Severe PE: 27	60	Serum IL-17 (ELISA)	Mean ± SD PE: 30.75 ± 1.97 weeks Mild PE: 30.45 ± 1.97 weeks Severe PE: 31.11 ± 1.973 weeks	Mean ± SD 30.11 ± 1.86 weeks	Mean ± SD PE: 8.81 ± 2.12 pg/ml Mild PE: 7.42 ± 1.23 pg/ml Severe PE: 10.51 ± 1.69 pg/ml	Mean ± SD 4.33 ± 0.68 pg/ml	PE versus healthy control: P < 0.001 Mild PE versus healthy control: P < 0.001 Severe PE versus healthy control: P < 0.001
Yu et al., 2017 [71]	China	Mild PE: 24 Severe PE: 22	24	Serum IL-17A (multiplex bead based assay)	Third trimester	Third trimester	#Median (25th - 75th percentile) Mild: 32.5 (23.0–46.6) pg/ml Severe: 35.2 (25.6–53.0) pg/ml	#Median (25th - 75th percentile) 16.4 (12.5–22.2) pg/ml	Mild PE vs. control: P < 0.05 Severe PE vs. control: P < 0.05
Perucci et al., 2023 [41]	Brazil	Total severe PE: 24 Severe EO-PE: 17 Severe LO-PE 7	34	Plasma IL-17A (ELISA)	Median (25th–75th percentiles) 32 (30–34) weeks	Median (25th–75th percentiles) 31 (30–35) weeks	Mean ± SD Total severe PE: 0.72 ± 0.30 ng/ml Severe EO-PE: 0.71 ± 0.29 ng/ml Severe LO-PE: 0.79 ± 0.25 ng/ml.	Mean ± SD 0.95 ± 0.26 ng/ml	Total severe PE vs. control: P = 0.021 Severe EO-PE vs. severe LO-PE: NS
Chen et al., 2019 [46]	China	29	27	Plasma IL-17 (ELISA)	Mean ± SD 37 ± 2.1 weeks (at term, CS delivery)	Mean ± SD 39 ± 2.2 weeks (at term, CS delivery)	#Mean ± SD 62.4 ± 49.0 pg/ml	#Mean ± SD 36.2 ± 27.7 pg/ml	P = 0.015
Chaiworapongsa et al., 2024 [72]	USA	96	213	Plasma IL-17-A (multiplex immunoassay)	Median (IQR) PE with normal angiogenic profile: 38.9 (37.9–39.7) weeks PE with abnormal angiogenic profile: 38.1 (37.6–39.0) weeks	Median (IQR) 39.1 (38.4–39.7) weeks	Median (IQR) PE with normal angiogenic profile: 0.5 (0.5–0.9) pg/ml PE with abnormal angiogenic profile: 0.6 (0.5–0.9) pg/ml	Median (IQR): 0.7 (0.5–1.0) pg/ml	PE with normal angiogenic profile vs. PE with abnormal angiogenic profile: P = 0.439. PE with normal angiogenic profile vs. Uncomplicated pregnancy: P = 0.149. PE with abnormal angiogenic profile vs. Uncomplicated pregnancy: P = 0.559
Charkiewicz et al., 2018 [73]	Poland	21	27	Plasma IL-17 (ELISA)	Median ± SD 33 ± 4.48 weeks	Median ± SD 34.2 ± 3.9 weeks	Mean ± SEM 395.24 ± 93.53 pg/ml	Mean ± SEM 456.37 ± 267.13 pg/ml	P = 0.1159
Hao et al., 2015 [74]	China	20	20	Plasma IL-17 (ELISA)	Mean ± SEM 36.5 ± 0.23 weeks (before CS delivery)	Mean ± SEM 37.8 ± 0.51 weeks (before CS delivery)	Mean ± SEM 85.89 ± 14.50 pg/ml	Mean ± SEM 53.64 ± 10.11 pg/ml	P < 0.05
Kaur et al., 2021 [75]	India	EO-PE with PTB: 199 (IL-17 measured in 191)	200 (IL-17 measured in 165)	Plasma IL-17 (ELISA)	Mean ± SEM 30.77 ± 0.21 weeks	Mean ± SEM 31.91 ± 0.28 weeks	#Mean ± SEM 71.4 ± 2.89 pg/ml	#Mean ± SEM 41.8 ± 1.33 pg/ml	P < 0.01
Kieffer et al., 2019 [76]	The Netherlands	15	15	Plasma IL-17A (multiplex bead based assay)	Median (IQR) 30.6 (27.3–31.9) weeks	Median (IQR) 30.6 (27.3–33.3) weeks	#Median (IQR) 7.835 (2.196)	#Median (IQR) 5.510 (3.582)	NS
Ozkan et al., 2013 [77]	Turkey	40	40	Plasma IL-17 (ELISA)	Median (min–max) 35 (24–40) weeks (prepartum)	Median (min–max) 38 (34–40) weeks (prepartum)	Median (min–max) 1.28 (1.08–4.24) pg/ml	Median (min–max) 1.5 (1–3.57) pg/ml	P < 0.01
Ribeiro et al., 2017 [78]	Brazil	EO-PE: 20 LO-PE:20	<34 weeks GA: 10 ≥34 weeks GA: 10	Plasma IL-17 (ELISA)	Median (range) EO-PE: 29 (26–33) weeks LO-PE: 37 (34–40) weeks	Median (range) <34 weeks GA: 30 (26–33) weeks ≥34 weeks GA: 37 (34–40) weeks	#Mean ± SD EO-PE: 18.6 ± 2.5 pg/ml LO-PE: 14.9 ± 2.2 pg/ml	#Mean ± SD <34 weeks GA: 7.85 ± 0.52 pg/ml ≥34 weeks GA: 8.16 ± 0.46 pg/ml	EO-PE vs LO-PE: P < 0.05 EO-PE vs. <34 weeks control: P < 0.05 LO-PE vs. ≥ 34 weeks control: P < 0.05
Zhang et al., 2017 [79]	China	Severe PE: 30	30	Plasma IL-17A (ELISA)	Mean ± SD 33.00 ± 2.95 weeks (before treatment)	Mean ± SD 33.00 ± 3.87 weeks	Mean ± SD 27.88 ± 5.05 pg/ml	Mean ± SD 17.91 ± 3.97 pg/ml	P < 0.001
Gharesi-Fard et al., 2016 [80]	Iran	15	15	Decidual and chorionic IL-17 (RT-qPCR)	Mean ± SEM 37.80 ± 0.49 weeks	Mean ± SEM 38.07 ± 0.22 weeks	#Mean ± SEM Decidua: 0.608 ± 0.160 a.u. Chorion: 0.907 ± 0.133 a.u.	#Mean ± SEM Decidua: 0.438 ± 0.118 a.u. Chorion: 0.790 ± 0.145 a.u.	Decidua: P = 0.2 Chorion: P = 0.6
**RPL (cases) vs. healthy pregnancy (controls)**									
Ding et al., 2023 [81]	China	108	108	Serum IL-17 (ELISA)	Median (IQR) 9.2 (8.0–10.0) weeks	Median (IQR) 9.1 (7.4–10.8) weeks	#Mean 35.182	#Mean 16.110	P < 0.001
Zhu et al., 2017 [82]	China	25	25	Serum IL-17A (ELISA)	Mean ± SD 44.14 + 5.83 days	Mean ± SD 46.48 + 4.66	# Mean ± SD 8.724 ± 0.969 pg/ml	Mean ± SD 8.070 ± 1.017 pg/ml	P = 0.03
Ziganshina et al., 2012 [83]	Russia	8	7	Serum IL-17A (Multiplex immunoassay)	5–6 weeks, 7–8 weeks, and 11–12 weeks	5–6 weeks, 7–8 weeks, and 11–12 weeks	Mean ± SEM Week 5–6: 1.54 ± 0.43 pg/ml Week 7–8: 1.93 ± 0.27 pg/ml Week 11–12:1.37 ± 0.004 pg/ml	Mean ± SEM Week 5–6: 1.56 ± 0.20 pg/ml Week 7–8: 1.37 ± 0.01 pg/ml Week 11–12: 1.64 ± 0.27 pg/ml	Week 5–6 uRPL versus healthy control: NS Week 7–8: uRPL versus healthy control: P < 0.05 Week 11–12 uRPL versus healthy control: NS Week 11–12 healthy control versus week 7–8 healthy control: P < 0.05
Khalaf et al., 2021 [39]	Egypt	40	40	Serum IL-17A (ELISA)	Between 7 and 10 weeks	Between week 7–10 weeks	#Mean ± SEM 26.1 ± 3.0 pg/ml	#Mean ± SEM 21.3 ± 2.0 pg/ml	NS
Cai et al., 2016 [84]	China	30	30	Decidual and serum IL-17 (ELISA)	Mean ± SD 50.90 ± 5.20 days	Mean ± SD 51.80 ± 5.03 days	#Mean ± SD Decidua: 32.5 ± 3.9 pg/ml Serum: 33.0 ± 3.7 pg/ml	#Mean ± SD Decidua: 17.3 ± 2.5 pg/ml Serum: 17.3 ± 2.5 pg/ml	Decidua: P < 0.05 Serum: P < 0.05
Li et al., 2017 [85]	China	30	30	Decidual IL-17 (ELISA)	7–9 weeks	7–9 weeks	Mean ± SD 320.85 ± 63.15 pg/ml	Mean ± SD 51.69 ± 51.72 pg/ml	P = 0.000
Qian et al., 2018 [86]	China	35	40	Decidual IL-17 (RT-qPCR and ELISA)	Mean ± SD 59.39 ± 1.82 days	Mean ± SD 58.12 ± 1.27 days	#Mean ± SEM mRNA: 3.8 0 ± 0.25 a.u. Protein: 74.8 ± 0 pg/ml	#Mean ± SEM mRNA: 3.12 ± 0.12 a.u. Protein: 66.0 ± 0 pg/ml	mRNA: NS Protein: NS
O'Hern Perfetto et al., 2015 [87]	US	9	11	Decidual IL-17 (RT-qPCR)	Mean ± SD 7.2 ± 1.5 weeks Median (range) 7 (6–10) weeks	Mean ± SD 7.6 ± 1.1 weeks Median (range) 8 (6–10) weeks	Median 0.0017 a.u.	Median 0.0012 a.u.	NS
Giannubilo et al., 2012 [88]	Italy	11	20	Trophoblastic IL-25 expression (RT-qPCR)	At abortion, <20 weeks GA	At abortion, <20 weeks GA	Median (IQR) (=ΔCt) 5.18 (4.46–5.76) a.u.	Median (IQR) (=ΔCt) 3.85 (3.6–4.51) a.u.	Not provided, "significant downregulation of IL-25 (2.5-fold reduction)"
Zhao et al., 2022 [89]	China	15	69	Serum IL-17 (multiplex microsphere-based cytometric assay)	Embryonic transfer day 0, 3, 6, 9, 16 (=5 weeks GA), 23 (=6 weeks GA), 30 (=7 weeks GA)	Embryonic transfer day 0, 3, 6, 9, 16 (=5 weeks GA), 23 (=6 weeks GA), 30 (=7 weeks GA)	Mean ± SEM Day 0: 46.64 ± 8.37 pg/ml Day 3: 56.84 ± 10.13 pg/ml Day 6: 56.82 ± 12.54 pg/ml Day 9: 50.58 ± 10.59 pg/ml Day 16: 50.60 ± 9.97 pg/ml Day 23: 53.20 ± 8.13 pg/ml Day 30: 57.06 ± 8.83 pg/ml	Mean ± SEM Day 0: 37.37 ± 3.48 pg/ml Day 3: 46.08 ± 3.89 pg/ml Day 6: 50.84 ± 5.50 pg/ml Day 9: 38.96 ± 3.64 pg/ml Day 16: 37.09 ± 3.24 pg/ml Day 23: 36.51 ± 3.34 pg/ml Day 30: 33.04 ± 3.11 pg/ml	Miscarriage, day 3 vs. day 0: P = 0.007 Live birth, day 3 vs. day 0: P = 0.0167 Live birth, day 6 vs. day 0: P = 0.037 Miscarriage vs. live birth, day 16: P = 0.026 Miscarriage vs. live birth, day 23: P = 0.032 Miscarriage vs. live birth, day 30: P = 0.0001
Ku et al., 2023 [90]	Singapore	17	97	Serum IL-17A (multiplex immunoassay)	Mean ± SD 6.4 ± 1.3 weeks	Mean ± SD 9.5 ± 1.0 weeks	Mean ± SD 6.78 ± 2.36 pg/ml	Mean ± SD 7.90 ± 1.80 pg/ml	P = 0.026
Nakashima et al., 2010 [91]	Japan	17	10	Decidual IL-17 expression (IHC)	7 weeks	7 weeks	Expression of IL-17 was detected in the stroma and blood vessels	No expression of IL-17 in the stroma and blood vessels	NA
Pongcharoen et al., 2007 [53]	Thailand	2	4	Placental IL-17 expression (IHC)	Not provided	Term	IL-17 was localized in cyto-, syncytio-, and extravillous trophoblast of SA placenta	NA	NA
**PTB (cases) vs. healthy term pregnancy (controls):**									
Shafiq et al., 2021 [106]	India	25	193	Serum IL-17A (multiplex immunoassay)	Median (IQR) 29.3 (28.5–30.4)	Median (IQR) 29.3 (28.5–30.4)	Median log2 transformed 2.56 pg/ml	Median log2 transformed 2.27 pg/ml	P = 0.008 Adjusted OR (95 % CI): 2.62 (1.11–6.17)
Bhati et al., 2023 [93]	India	160	160	Placental IL-17 (RT-qPCR)	<37 weeks	>37 weeks	Relative fold change: Infected sPTB: 4.687 Uninfected sPTB:2.283	Relative fold change: Term controls:0.975	Uninfected sPTB vs. Infectd PTB: P < 0.001 Uninfected sPTB vs. Term controls: P < 0.001 infected sPTB vs. Term controls: P < 0.001
Son et al., 2015 [94]	Korea	Total cervical insufficiency: 67 Very PTB: 26 Late PTB: 12 Term birth: 29	10	Amniotic fluid IL-17A (multiplex magnetic bead immunoassay)	Median (IQR) Very PTB: 21.7 (19.5–22.5) weeks Late PTB: 22.2 (19.9–24.3) weeks Term birth: 22.0 (20.7–23.2) weeks	Mean ± ? 19.2 ± 0.2 weeks	Median (IQR) Total cervical insufficiency: 86.3 (33.7–139.1) pg/ml Very PTB: 135.1 (86.6–188.9) pg/ml Late PTB: 82.3 (38.8–106.2) pg/ml Term birth: 65.2 (43.8–105.9) pg/ml	Median (IQR) 12.5 (3.5–22.1) pg/ml	Total cervical insufficiency vs. controls: P < 0.001 Very PTB vs. late PTB: P < 0.05 Very PTB vs. cervical insufficiency term birth: P < 0.05 *Correlation IL-17A and GA at delivery: R=-0.522, P<0.001*
Buxton et al., 2021 [95]	Mexico	12	78	Cervico-vaginal IL-17 (multiplex magnetic bead assay)	First, second and third trimester	First, second and third trimester	Median First trimester: 3.20 pg/ml Second trimester: 3.39 pg/ml Third trimester: 3.20 pg/ml Entire pregnancy average: 2.74 pg/ml	Median First trimester: 1.41 pg/ml Second trimester: 2.61 pg/ml Third trimester: 2.72 pg/ml Entire pregnancy average: 2.62 pg/ml	All NS OR (95%CI) first trimester: 1.39 (1.04, 1.86) OR (95%CI) second trimester: 1.30 (0.98, 1.74) OR (95%CI) third trimester: 1.35 (0.99, 1.85) OR (95%CI) entire pregnancy: 1.38 (1.01, 1.89)

NA: not applicable. IHC: Immunohistochemistry. RT-qPCR: Quantitative reverse transcription polymerase chain reaction. ELISA: enzyme-linked immunosorbent assay. SEM: standard error of the mean. NS: not significant. SD: standard deviation. IQR interquartile range. CS: caesarian section. PE: preeclampsia. EO- and LO-PE: early- and late-onset preeclampsia. GH: gestational hypertension. HELLP: hemolysis, elevated liver enzymes and low platelets. GA: gestational age. FGR: fetal growth restriction: PTB: preterm birth. a.u.: arbitrary units. SA: spontaneous abortion. urPL: unexplained recurrent miscarriage. OR: odds ratio.

#: values extracted with WebPlotDigitizer.

∗Measures below detection limit.

**Table:** 

**Study**	**Location**	**Case population size**	**Control population size**	**Outcome measures (technique)**	**Gestational age at measurement of cases**	**Gestational age at measurement of controls**	**IL-17 level cases group**	**IL-17 level control group**	**P-value**
**Healthy pregnancy (cases): vs. non-pregnancy (controls):**									
Uz et al., 2010 [96]	USA	8	NA	Placental IL-23 (IHC)	First trimester	NA	IL-23 is expressed in decidual cells, glandular epithelium, cyto- syncytio- and extravillous trophoblasts	NA	NA
Poordast et al., 2017 [45]	Iran	30	30	Serum IL-23 (ELISA)	Third trimester (before treatment)	NA	#Mean ± SEM 750 ± 44 pg/ml	#Mean ± SEM 952 ± 44 pg/ml	P = 0.002
Tagoma et al., 2019 [43]	Estonia	115	42	Plasma IL-23 (multiplex magnetic bead assay)	Mean ± SD 26.2 ± 1.6 weeks	NA	Median (25‐75th percentiles): 144.8 (31.0–291.6) pg/ml	Median (25‐75th percentiles): 9.0 (3.5–81.4) pg/ml	Not provided
Lang et al., 2021 [44]	China	102	147	Serum IL-23 (ELISA)	Third trimester	NA	#Mean ± SEM 34.9 ± 10.8 pg/ml	#Mean ± SEM 35.7 ± 10.6 pg/ml	Not provided
**B: PE pregnancy (cases) vs. healthy pregnancy (controls):**									
Lang et al., 2021 [44]	China	115	102	Serum IL-23 (ELISA)	Third trimester	Third trimester	#Mean ± SEM 37.0 ± 10.9 pg/ml	#Mean ± SEM 34.9 ± 10.8 pg/ml	Not provided
Poordast et al., 2017 [45]	Iran	30	30	Serum IL-23 (ELISA)	Third trimester (before treatment)	Third trimester	#Mean ± SEM 800 ± 49 pg/ml	#Mean ± SEM 750 ± 44 pg/ml	NS
Darmochwal-Kolarz et al., 2017 [65]	Poland	34 (PE-associated FGR)	35	Serum IL-23 (immunoenzymatic assay)	32.05 ± 2.14 weeks	32.62 ± 1.63 weeks	Median (IQR): 1.93 (1.37–2.68) pg/ml	Median (IQR): 1.95 (1.11–2.84) pg/ml	NS
Eghbal-Fard et al., 2019 [66]	Iran	50	50	Serum IL-23 (ELISA)	Mean ± SD: 35.8 ± 5.0 weeks	Mean ± SD: 37.1 ± 2.3 weeks	Mean ± SD: 33.15 ± 15.95 pg/ml	Mean ± SD: 25.30 ± 12.47 pg/ml	NS
Forghani et al., 2023 [97]	Iran	Total PE: 75 Severe PE: 46 Mild PE: 29 EO-PE: 34 LO-PE: 41	35	Serum IL-23 (ELISA)	Mean ± SD: 33.71 ± 5.36 weeks	Mean ± SD: 33.35 ± 3.89 weeks	Mean ± SD: Total PE: 24.461 ± 8.027 pg/ml Mild PE: 20.448 ± 6.363 pg/ml Severe PE: 26.991 ± 7.989 pg/ml EO-PE: 29.532 ± 7.192 pg/ml LO-PE: 20.256 ± 6.044 pg/ml	Mean ± SD: 17.622 ± 6.310 pg/ml	Total PE vs. controls: P < 0.001 Severe PE vs. controls: P < 0.001 Mild PE vs. controls: P = 0.08 Severe vs. mild PE: P < 0.001 EO-PE vs. LO-PE: P < 0.001
Gharesi-Fard et al., 2016 [80]	Iran	15	15	Decidual and chorionic IL-23 (RT-qPCR)	Mean ± SEM 37.80 ± 0.49 weeks (CS delivery)	Mean ± SEM 38.07 ± 0.22 weeks (CS delivery)	#Mean ± SEM Decidua: 0.660 ± 0.360 a.u. Chorion: 1.83 ± 0.17 a.u.	#Mean ± SEM Decidua: 0.573 ± 0.130 a.u. Chorion: 1.06 ± 0.10 a.u.	Decidua: P = 0.4 Chorion: P = 0.02
Kieffer et al., 2020 [98]	The Netherlands	EO-PE: 6 (100 % CS delivery) LO-PE: 8 (25 % CS delivery)	15 (33.3 % CS delivery)	Decidua parietalis and basalis IL-23 (RT-qPCR)	Median (IQR) EO-PE: 30.65 (29.29–31.32) weeks LO-PE: 38.14 (37.14–38.79) weeks	Median (IQR) 39.29 (39.0–39.86) weeks	#Median Parietalis, EO-PE: 0.00339 a.u. Basalis, EO-PE: 0.000908 a.u. Parietalis, LO-PE: 0.00356 a.u. Basalis, LO-PE: 0.00103 a.u.	#Median Parietalis: 0.00252 a.u. Basalis: 0.000232 a.u.	Decidua parietalis, EO-PE vs. control: NS Decidua parietalis, LO-PE vs. control: NS Decidua basalis, EO-PE vs. control: P = 0.030 Decidua basalis, LO-PE vs. control: P = 0.011
**RPL (cases) vs. elective healthy pregnancy termination (controls):**									
Cai et al., 2016 [84]	China	30	30	Decidual and serum IL-23 (ELISA)	Mean ± SD 50.90 ± 5.20 days	Mean ± SD 51.80 ± 5.03 days	#Mean ± SD Decidua: 27.9 ± 3.8 pg/ml Serum: 27.9 ± 4.0 pg/ml	#Mean ± SD Decidua: 16.5 ± 2.7 pg/ml Serum: 17.3 ± 3.1 pg/ml	Decidua: P < 0.05 Serum: P < 0.05
Ding et al., 2023 [81]	China	108	108	Serum IL-23 (ELISA)	Median (IQR) 9.2 (8.0–10.0) weeks	Median (IQR) 9.1 (7.4–10.8) weeks	#Mean 32.98 pg/ml	#Mean 22.28 pg/ml	P < 0.001
Li et al., 2017 [85]	China	30	30	Decidual IL-23 (ELISA)	7–9 weeks	7–9 weeks	Mean ± SD: 156.73 ± 57.21 pg/ml	Mean ± SD: 124.14 ± 36.60 pg/ml	P = 0.011
Qian et al., 2018 [86]	China	35	40	Decidual IL-23 (RT-qPCR and ELISA)	Mean ± SD 59.39 ± 1.82 days	Mean ± SD 58.12 ± 1.27 days	#Mean ± SEM mRNA: 1.92 ± 0.05 a.u. Protein: 113 ± 5 pg/ml	#Mean ± SEM mRNA: 1.73 ± 0.05 a.u. Protein: 76.8 ± 0 pg/ml	mRNA: NS Protein: P < 0.05
O'Hern Perfetto et al., 2015 [87]	US	9	11	Decidual IL-23 (RT-qPCR)	Mean ± SD 7.2 ± 1.5 weeks Median (range) 7 (6–10) weeks	Mean ± SD 7.6 ± 1.1 weeks; Median (range) 8 (6–10) weeks =	Median: 0.0017 a.u.	Median: 0.0026 a.u.	NS

NA: not applicable. IHC: immunohistochemistry. ELISA: enzyme-linked immunosorbent assay. SEM: standard error of the mean. PE: preeclampsia. NS: not significant. FGR: fetal growth restriction. IQR: interquartile range. SD: standard deviation. EO- and LO-PE: early- and late-onset preeclampsia. RT-qPCR: Quantitative reverse transcription polymerase chain reaction. CS: caesarian section. uRPL: unexplained recurrent miscarriage.

#: values extracted with WebPlotDigitizer.

## Data Availability

Data will be made available on request.
